# Systematic Identification of *cis*-Regulatory Sequences Active in Mouse and Human Embryonic Stem Cells

**DOI:** 10.1371/journal.pgen.0030145

**Published:** 2007-08-31

**Authors:** Marica Grskovic, Christina Chaivorapol, Alexandre Gaspar-Maia, Hao Li, Miguel Ramalho-Santos

**Affiliations:** 1 Institute for Regeneration Medicine, University of California San Francisco, San Francisco, California, United States of America; 2 Diabetes Center, University of California San Francisco, San Francisco, California, United States of America; 3 Department of Biochemistry and Biophysics, University of California San Francisco, San Francisco, California, United States of America; 4 California Institute for Quantitative Biomedical Research, University of California San Francisco, San Francisco, California, United States of America; 5 Graduate Program in Biological and Medical Informatics; University of California San Francisco, San Francisco, California, United States of America; 6 Doctoral Program in Biomedicine and Experimental Biology, Center for Neuroscience and Cell Biology, University of Coimbra, Coimbra, Portugal; University of Cambridge, afsmith@mole.bio.cam.ac.uk

## Abstract

Understanding the transcriptional regulation of pluripotent cells is of fundamental interest and will greatly inform efforts aimed at directing differentiation of embryonic stem (ES) cells or reprogramming somatic cells. We first analyzed the transcriptional profiles of mouse ES cells and primordial germ cells and identified genes upregulated in pluripotent cells both in vitro and in vivo. These genes are enriched for roles in transcription, chromatin remodeling, cell cycle, and DNA repair. We developed a novel computational algorithm, CompMoby, which combines analyses of sequences both aligned and non-aligned between different genomes with a probabilistic segmentation model to systematically predict short DNA motifs that regulate gene expression. CompMoby was used to identify conserved overrepresented motifs in genes upregulated in pluripotent cells. We show that the motifs are preferentially active in undifferentiated mouse ES and embryonic germ cells in a sequence-specific manner, and that they can act as enhancers in the context of an endogenous promoter. Importantly, the activity of the motifs is conserved in human ES cells. We further show that the transcription factor NF-Y specifically binds to one of the motifs, is differentially expressed during ES cell differentiation, and is required for ES cell proliferation. This study provides novel insights into the transcriptional regulatory networks of pluripotent cells. Our results suggest that this systematic approach can be broadly applied to understanding transcriptional networks in mammalian species.

## Author Summary

Embryonic stem cells have two remarkable properties: they can proliferate very rapidly, and they can give rise to all of the body's cell types. Understanding how gene activity is regulated in embryonic stem cells will be an important step towards therapeutic applications. The activity of genes is regulated by proteins called transcription factors, which bind to stretches of DNA sequences that act as on or off switches. We identified genes that are active in mouse embryonic stem cells but not in differentiated cells. We reasoned that if these genes have similar patterns of activity, they may be regulated by the same transcription factors. We therefore developed a computational approach that takes information on gene activity and predicts DNA sequences that may act as switches. Using this approach, we discovered new DNA switches that regulate gene activity in mouse and human embryonic stem cells. Furthermore, we identified a transcription factor that binds to one of these DNA switches and is important for the rapid proliferation of embryonic stem cells. Our approach sheds light on the genetic regulation of embryonic stem cells and will be broadly applicable to questions of how gene activity is regulated in other cell types of interest.

## Introduction

Pluripotent stem cells can give rise to all fetal and adult cell lineages, including the germline. The prototypical pluripotent stem cells are embryonic stem (ES) cells [1,2]. ES cells are a remarkable model for the study of early development and hold promise as a source for cell replacement therapies [3]. To successfully manipulate ES cells in culture, it is important to understand the mechanisms by which ES cells maintain their self renewal and pluripotency.

ES cells are derived from the inner cell mass of the blastocyst, a group of cells that gives rise to all cells of the fetus. After the blastocyst implants in the uterus and gastrulation ensues, most cells of the embryo lose the ability to give rise to pluripotent stem cells, except for primordial germ cells (PGCs) [4,5]. PGCs are the germline precursors that give rise to sperm or eggs. When cultured in vitro, PGCs give rise to embryonic germ (EG) cells, pluripotent stem cells very similar to ES cells [6,7].

Several regulatory pathways that control ES cell pluripotency and self renewal have recently been identified (reviewed in [8]). Factors involved include the leukemia inhibitory factor (LIF) and BMP signaling pathways [9–12], and transcription factors Nanog [13,14] and Oct4 [15,16]. Interestingly, the signaling pathways do not appear to be conserved between mouse and human ES cells [17–20], but the transcriptional regulators Oct4 and Nanog are required in ES cells of both species [21–23]. Recent studies indicate that transcription factors other than Oct4 and Nanog are also important for maintenance of the ES cell state [24,25]. A major goal will be to obtain a complete description of the transcriptional regulatory networks of ES cells.

The increasing availability of whole genome sequences and high-throughput experimental methods, such as microarrays, have led to the development of systematic approaches for deciphering transcriptional regulation. Such analyses generally lead to the identification of sets of genes whose expression is coregulated. It has been shown that genes within a coregulated set often share common *cis*-regulatory motifs, corresponding to transcription factor binding sites, in their upstream genomic sequences (for reviews see [26,27]). A number of computational algorithms have been developed to identify such regulatory motifs. These algorithms include enumeration of overrepresented substrings or regular expression patterns, local multiple sequence alignment, or sequence segmentation to decompose the DNA sequence into the most plausible set of motifs [28–32]. The strategy of identifying clusters of coregulated genes by expression profiling followed by a computational search for regulatory motifs has been successfully applied to a number of questions, mostly in lower eukaryotes such as yeast. For mammalian species, the problem is much more challenging [33], as the genomes are more complex and regulation often involves combinatorial action of transcription factors [34]. Examples of computational de novo motif discovery followed by experimental validation in mammalian species are scarce. One experimentally validated case recently reported using motif discovery to identify mouse transcription factors that regulate oxidative phosphorylation [35].

Recent algorithms targeted at higher eukaryotes use interspecies comparisons to identify functional motifs in orthologous promoters [36–38], because functional elements are subject to selective pressure and tend to evolve more slowly than nonfunctional sequences [34]. These algorithms typically use conserved blocks of DNA sequence that can be aligned to reduce the background noise. However, alignment-based approaches can miss important sequence motifs as many regulatory sequences do not fall into conserved regions [39,40].

In this paper, we used a combination of gene expression profiling with computational genomic analyses and biochemical assays to systematically identify novel *cis*-regulatory sequences that control gene expression in pluripotent stem cells. To gain insight into the transcriptional regulatory networks of pluripotent cells, we compared the gene expression profiles of ES cells and PGCs to embryonic and adult somatic cell types. We identified clusters of genes upregulated in ES cells and PGCs, which include several known markers of pluripotency. To identify regulatory motifs that control gene expression within these clusters, we developed a novel algorithm, CompMoby. This algorithm combines the strategies of comparative genomics with DNA sequence segmentation to identify sets of motifs in the upstream regions of coregulated genes. Using CompMoby, we identified motifs that are statistically overrepresented in genes upregulated in pluripotent cells and highly conserved across multiple mammalian species. We demonstrate that several of the predicted motifs are novel regulatory elements of gene expression in mouse and human ES cells. Finally, we show that the transcription factor NF-Y binds to one of the motifs, is differentially expressed during ES cell differentiation, and is required for ES cell proliferation.

## Results

### Genes Upregulated in Pluripotent Cells Are Involved in Transcription, Chromatin Remodeling, Cell Cycle, and DNA Repair

**Figure 1:**
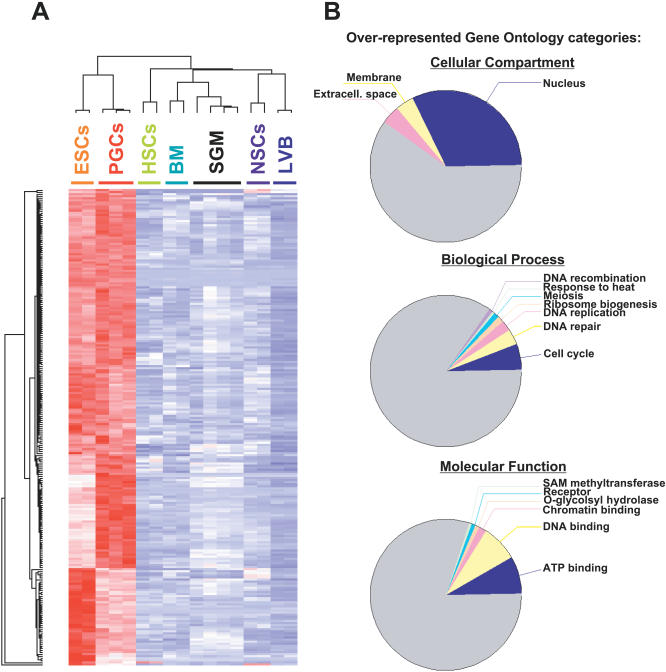
Identification of Genes Upregulated in Pluripotent Cells (A) Gene expression profiling of ES cells and PGCs. Hierarchical clustering was used to identify genes upregulated in ES cells and PGCs relative to SGMs, bone marrow (BM), hematopoietic stem cells (HSCs), lateral ventricles of the brain (LVB), and neural stem/progenitor cells (NSCs) [42]. The cluster shown depicts gene expression of 230 probe sets upregulated in ESCs and PGCs. Red means the gene is upregulated, blue means it is downregulated. (B) Functional annotation of genes upregulated in ES cells and PGCs. Gene Ontology analysis was performed to identify functional categories overrepresented in the cluster of genes upregulated in pluripotent cells (A). The top pie chart represents the ontology “cellular component,” the middle pie chart represents the ontology “biological process,” and the bottom pie chart represents the ontology “molecular function.” Categories shown are significantly overrepresented at *p* < 0.005. Grey slices represent categories with *p* > 0.005.

The identification of the gene expression profiles of PGCs and neighboring somatic cells of the genital ridge/mesonephros area (SGM) is described elsewhere (Wei et al., submitted). Briefly, PGCs and SGM cells were isolated by fluorescence-activated cell sorting from 11.5-d post coitum (dpc) mouse embryos carrying the Oct4/EGFP transgene. This construct has been shown to drive expression of EGFP specifically in PGCs [41]. We then identified the gene expression profiles of PGCs and SGM cells using Affymetrix U74Av2 microarrays. The raw data can be obtained from ArrayExpress. The complete normalized expression data can be found in Dataset S1. We compared the gene expression profiles of PGCs and SGM cells to those of embryonic and adult stem cells, and adult differentiated tissues [42]. Hierarchical clustering revealed similarities at the gene expression level between PGCs and ES cells (Figure 1A). Furthermore, the transcription profile of ES cells is more similar to PGCs than to that of adult stem cells.

This result suggests that aspects of the transcriptional regulation of pluripotency of ES cells are maintained in PGCs during embryogenesis. We therefore sought to identify clusters of genes upregulated in ES cells and PGCs, but not in other cell types. Figure 1A depicts a composite cluster of 230 probe sets upregulated (in red) in pluripotent cells, and downregulated or not expressed (in blue) in adult stem cells and differentiated cells (Figure 1A and Dataset S2). These genes are also largely downregulated upon differentiation of ES cells, a further indication that their expression correlates with the pluripotent state (H. Chipperfield, S. Zhong, D. Melton, and W. Wong, personal communication). This cluster includes several known markers of pluripotency (see below).

We used Onto-Express [43] to search the Gene Ontology database for functional categories overrepresented in the cluster of genes upregulated in pluripotent cells (Figure 1B). The full list of Gene Ontology categories can be found in Datasets S3, S4, S5, and S6. Overall, our data indicate that pluripotent cells are highly enriched for nuclear activities related to cell cycle, DNA repair, transcription, and chromatin remodeling.

### Computational Identification of Putative Regulatory Motifs

Genes coexpressed in pluripotent cells may be (at least in part) coregulated by the same transcription factors. It follows that transcription factor binding sites responsible for driving gene expression in pluripotent cells are likely to be overrepresented in the *cis*-acting regions of those genes. We therefore took a computational approach to identify DNA motifs that are statistically overrepresented in the putative promoter and enhancer regions of genes upregulated in pluripotent cells. To reduce noise in our computational analysis, we derived a smaller subset of genes, those with highly significant changes in expression (standard deviation/mean > 0.6). A smaller cluster of 55 probe sets (Dataset S7) was obtained that includes several known markers of pluripotency, such as Oct4, Nanog, Gdf3, Dppa2, Esg1, Utf1, and Tera.

**Figure 2:**
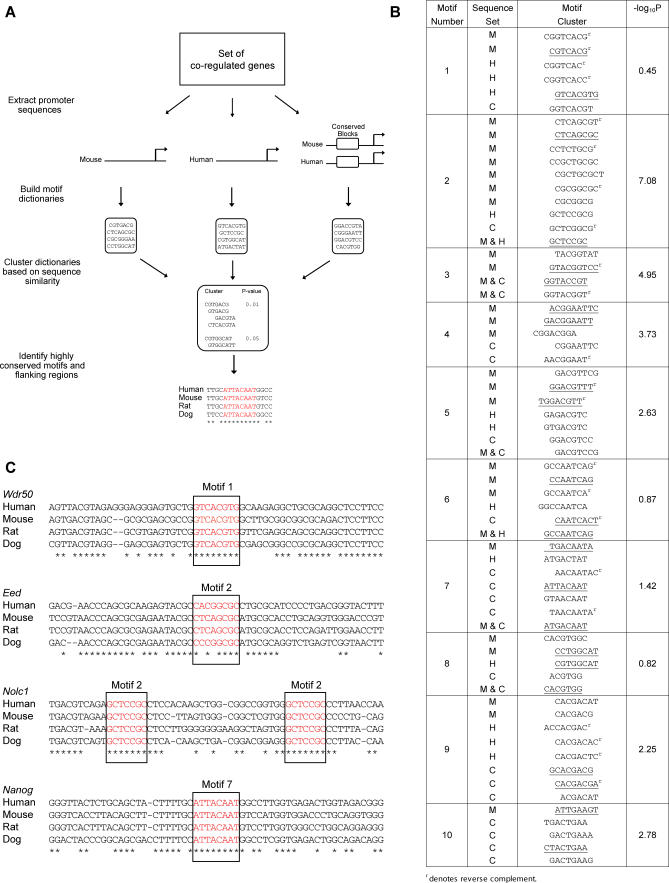
Computational Analysis and Identification of Regulatory Motifs (A) Schematic diagram of the CompMoby algorithm. (B) Top ten predicted motif clusters from CompMoby analysis of 2,000-bp sequences upstream from the transcriptional start site of 55 probe sets upregulated in pluripotent cells. Upstream sequence sets are given as Mouse (M), Human (H), and Conserved blocks between mouse and human (C). The fourth column lists −log_10_ *p*-values, which are calculated based on overrepresentation of the motif cluster in sequences upstream of the 55 probe sets relative to all other probe sets. *p*-Values are Bonferroni corrected for multiple testing. Underlined motifs were chosen for experimental characterization. (C) Examples of motifs (red) found by CompMoby to be highly conserved across four mammalian species. Asterisks denote bases conserved across all four species.

To identify *cis*-regulatory motifs involved in transcriptional regulation of pluripotency-associated genes, we developed CompMoby (Comparative MobyDick), which improves upon MobyDick [28] by incorporating a flexible analysis of evolutionary conservation (Figure 2A). From a set of coregulated genes, CompMoby builds multiple dictionaries (lists of motifs) from the upstream noncoding sequences of individual genomes as well as sequences conserved across species. The motifs of these dictionaries are clustered to obtain a final dictionary of motif clusters. CompMoby then screens for motif clusters that are overrepresented in the set of coregulated genes compared to the entire genome.

Functional elements may not reside within conserved regions [39,40], and an advantage of CompMoby is that it does not solely rely on sequence alignments, but also uses information from individual genomes. By combining these two sets of information, CompMoby can identify conserved sites that are aligned, sites that are conserved but not aligned, and nonconserved sites. CompMoby is flexible; motifs do not have to be exactly conserved across species, since clustering the multiple dictionaries derived from different sets of sequences will group motifs related to each other by a few mutations.

We employed CompMoby to identify putative *cis*-acting motifs in the upstream sequences that may be shared among upregulated genes in mouse pluripotent cells and their human orthologs (Datasets S8–S13). From our final dictionary (Figure 2B and Datasets S14 and S15), we selected ten motif clusters and used promoter alignment data between human, mouse, rat, and dog [44] to systematically identify highly conserved motifs and their flanking regions (Figure 2C and Dataset S16) within the promoters of genes upregulated in pluripotent cells. We chose 25 different motifs and their flanking regions from our top ten motif clusters for further experimental characterization (underlined sequences in Figure 2B).

It is important to note that two of the predicted motifs correspond to putative binding sites of known transcriptional regulators of ES cells. The motif 7 cluster contains the sequence ATTACAAT, which has been implicated in Sox2 binding [45]. This sequence and its flanking regions are conserved in the upstream sequences of the Nanog gene in human, mouse, rat, and dog (Figure 2C). Interestingly, the conserved sequence corresponds to the recently described binding site for Oct4 and Sox2 that is required for *Nanog* expression [45,46], indicating that we have indeed identified a functional motif that regulates a pluripotency-associated gene. Cluster 8 contains a palindromic motif that matches the known canonical binding motif for Myc [47,48]*.* Although several other members of the basic helix-loop-helix family can bind this motif [49], it is interesting to note that c-Myc has recently been implicated in the regulation of self-renewal and pluripotency in mouse ES cells [50], and that it is part of a cocktail of factors capable of inducing pluripotency [51]. These results demonstrated the power of CombMoby and suggested that the other novel identified motifs may also be functional.

### Identification of Novel Regulatory Motifs in Mouse ES Cells

**Figure 3:**
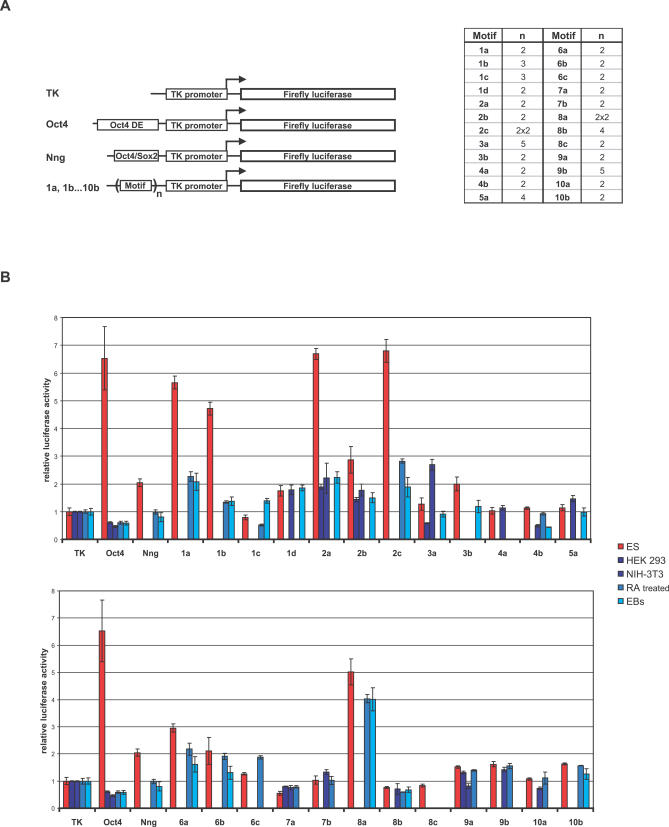
Experimental Validation of Predicted Regulatory Motifs (A) Schematic representation of constructs used in this study. A fragment of the *Oct4* distal enhancer (DE, bp −2,181 to −1,939) (Oct4), and the Oct4/Sox2 regulatory motif identified in cluster 7 and comprising a part of the *Nanog* promoter (bp −190 to −156) (Nng) were used as positive controls. Predicted motif sequences (1a, 1b, …, 10b) were fused to a construct containing Firefly luciferase driven by a minimal TK promoter. The number of repeats for a given motif is indicated in the right panel. Constructs 2c and 8a bear two repeats of genomic sequence that each contains two predicted regulatory motifs. (B) Regulatory activity of predicted motifs in mouse ES cells. Constructs described in (A) were transfected in undifferentiated mouse ES cells (red bars); or HEK293, NIH-3T3, and ES cells differentiated by formation of EBs or addition of RA (blue bars). A plasmid containing Renilla luciferase driven by the TK promoter was cotransfected. After 24 h, cells were lysed and assayed for luciferase activities. Firefly values were corrected for Renilla values, and the activities of the tested constructs were calculated relative to the activity of the TK construct, which was set to 1. For undifferentiated mouse ES cells, each construct was tested in several independent experiments (five on average), and representative results are shown. For HEK293, NIH-3T3, EB-derived, and RA-differentiated ES cells, one representative experiment is shown for each (blue bars). Bars represent averages of triplicates performed in each single experiment. Error bars depict standard deviation.

We next sought to assess the transcriptional regulatory activity of the predicted motifs. We transfected mouse ES cells with Firefly luciferase reporter constructs containing the motifs upstream of a heterologous thymidine kinase (TK) minimal promoter (Figure 3). Each construct contained a motif and its flanking sequences (median length 30 bp) present in at least two repeats (table in Figure 3A; Dataset S18). As a positive control, we used a 242-bp fragment of the *Oct4* distal enhancer (DE) (Oct4, Figure 3A). Since one of our predicted motifs together with its flanking sequence has already been shown to regulate Nanog and be sufficient for gene expression in ES cells [45,46], we used it as an additional positive control (Nng, Figure 3A). Both controls contain an Oct4/Sox2 binding site, the only known enhancer element shown to specifically regulate expression of several genes preferentially expressed in ES cells [45,52–56].

When compared to the construct containing only the TK promoter (Figure 3A), 14 out of 25 motif-containing constructs showed a change in luciferase expression in transfected ES cells (Figure 3B, red bars), suggesting that the predicted motifs are functional enhancers of transcription. Notably, the enhancer activity of several motifs was higher than the activity of the previously identified Oct4/Sox2 enhancer (Figure 3B, compare motifs 1a, 1b, 2a, and 2c to Nng).

For some motifs, we tested different numbers of repeats (Figure 3A and Figure S1) and found that the increase in luciferase expression was directly proportional to the number of repeats, further suggesting that the predicted motifs act as transcriptional activators. To determine if the observed transcriptional activation is specific for pluripotent ES cells, we transfected several differentiated cell types: HEK293, NIH-3T3, and ES cells differentiated either by formation of embryoid bodies (EBs) or addition of retinoic acid (RA) (Figure 3B, blue bars). Several motifs showed decreased activity in differentiated cells compared to ES cells, indicating that they are preferentially active as transcriptional enhancers in pluripotent ES cells.

Two of the tested motifs appear to have repressing activities. While showing little activity in ES cells, motif 4b seems to downregulate expression in NIH 3T3s (which are transformed mouse embryonic fibroblasts) and EB cells (Figure 3B). Therefore, motif 4b might bind a repressor necessary for downregulation of genes expressed in ES cells upon differentiation. In contrast, motif 7a appears to confer repression preferentially in ES cells, suggesting that it may control the levels of ES cell-expressed genes (Figure 3B). In summary, we have identified several novel *cis*-acting motifs that are sufficient to regulate gene expression preferentially in undifferentiated mouse ES cells. These results demonstrate that CompMoby can successfully predict functional motifs in mammals and even compares favorably to studies in organisms with simpler genomes such as yeast [57].

**Figure 4:**
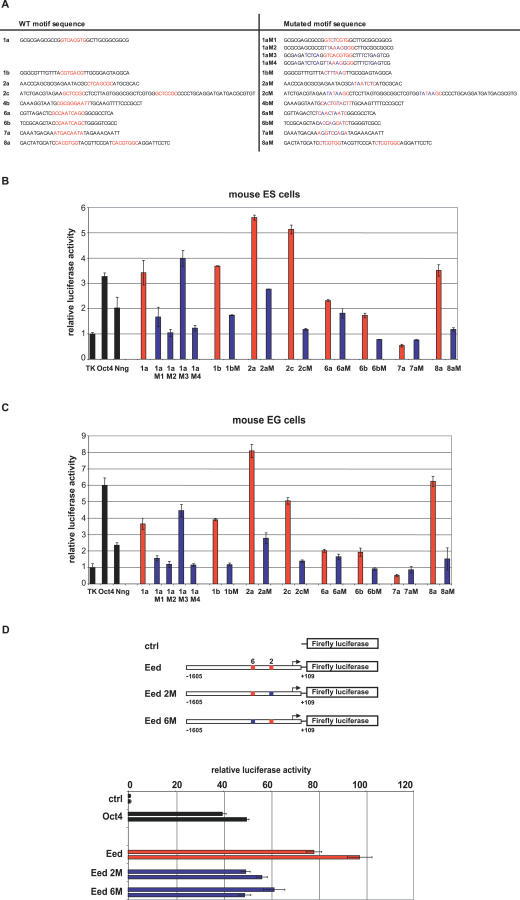
The Predicted Regulatory Motifs Are Sequence-Specific, Active in Both Mouse ES and EG Cells, and Act as Functional Enhancers of the Endogenous Eed Promoter (A) Mutations in the predicted regulatory motifs. Sequences containing the predicted regulatory motifs (depicted in red) flanked by endogenous sequences are shown on the left. Sequences containing point mutations (depicted in blue) are shown on the right. (B) Regulatory activity of the predicted motifs and their mutated counterparts in mouse ES cells. Data were collected and analyzed as described in Figure 3B. Representative results from five independent experiments are shown. Bars represent averages of triplicates performed in each single experiment. Error bars depict standard deviation. Wild-type sequences, red bars; mutated sequences, blue bars. (C) Regulatory activity of predicted motifs and their mutated counterparts in mouse EG cells. Data were collected and analyzed as described in Figure 3B. Representative results from two independent experiments are shown. Bars represent averages of triplicates performed in each single experiment, error bars depict standard deviation. Wild-type sequences, red bars; mutated sequences, blue bars. (D) Activity of regulatory motifs 2 and 6 present in Eed upstream genomic sequence. A 1.7-kb fragment of Eed upstream genomic sequence (bp −1,605 to +109 relative to the transcription start site) was cloned and fused to the Firefly luciferase reporter gene (Eed). Mouse ES cells were transfected, and the activity of the Eed construct was compared to the activities of the construct containing luciferase reporter gene alone (ctrl), a TK-bearing construct containing the Oct4 DE (Oct4), an Eed construct containing four point mutations in motif 2 (Eed 2M), and an Eed construct containing four point mutations in motif 6 (Eed 6M). Data were collected and analyzed as described in Figure 3B. Results from two independent experiments are shown. Bars represent averages of triplicates performed in each single experiment; error bars depict standard deviation. Wild-type sequences, red bars; mutated sequences, blue bars. The same mutations also significantly reduce activity of the Eed promoter in EG cells (unpublished data).

We decided to focus on eight motifs that showed the most interesting levels of activity in undifferentiated versus differentiated cells (Figure 4A). To confirm that the predicted motifs are indeed responsible for this activity, we performed mutational analyses by introducing point mutations at every second position of a motif sequence, with the exception of motif 1a and 8a. Motifs 1a and 8a, while clearly belonging to distinct motif clusters, have the sequence CACGTG in common (Figures 2B and 4A). CACGTG has been previously identified as a binding site for c-Myc and several other transcription factors of the basic helix-loop-helix family (see above), and a point mutation in CACGTG inhibits binding of Myc proteins [58]. Therefore, we decided to introduce a single point mutation in motifs 1a and 8a (1aM1, and 8aM, respectively). When transfected into mouse ES cells (Figure 4B), both mutated motifs 1a and 8a showed a drastic reduction in activity. This result indicates that their enhancer activity is regulated by the CACGTG sequence, likely through the binding of a basic helix-loop-helix transcription factor. The construct tested for motif 1a contains GC-rich sequences in the regions flanking the predicted motif. Particular GC-rich sequences may be bound by the Sp1 transcription factor [59]. To test the contribution of flanking sequences to the activity of motif 1a, we introduced four point mutations in each of the flanking regions (1aM3), four point mutations in the motif 1a sequence (1aM2), and both combined (1aM4). Our results indicate that the enhancing activity of motif 1a is due to the predicted motif sequence, and not to the flanking regions (Figure 4B). Similarly, the activity of all other motifs was significantly reduced or abolished when mutated, indicating that the predicted motif sequences are responsible for their activity.

To determine whether the identified motifs are specifically active in ES cells only, we tested their activity in another pluripotent cell type, EG cells. We found that all of the tested motifs have comparable levels of activity in both pluripotent cell types (Figure 4C). Next, we investigated whether any of the motifs are required for regulation of gene expression in the context of an endogenous promoter. One of the genes we identified as upregulated in pluripotent cells is *Eed* (Datasets S2 and S7), a component of chromatin remodeling complexes that regulate transcriptional silencing in ES cells [60]. The regulatory sequences of the *Eed* gene have not been described, and the genomic sequence upstream of *Eed* contains several of our predicted motifs. Point mutations in the sequences representing predicted motifs 2 and 6 significantly reduced the activity of the *Eed* promoter (Figure 4D), indicating that motifs 2 and 6 are necessary for maximal expression driven by the endogenous *Eed* promoter. Future studies will be necessary for complete dissection of the *Eed* promoter. Nevertheless, our proof-of principle experiments demonstrate that at least some of the motifs identified as sufficient to enhance transcription of a heterologous promoter in pluripotent stem cells are also functional enhancers of an endogenous promoter. In summary, we have identified novel, bona fide regulatory motifs present in genes preferentially expressed in pluripotent cells. We anticipate that our approach will greatly accelerate the dissection of enhancer/promoter elements of pluripotency-associated genes.

### The Identified Regulatory Motifs Are Active in Human ES Cells

**Figure 5:**
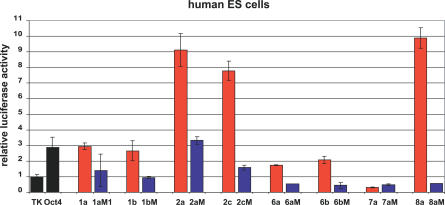
The Activity of the Identified Motifs Is Conserved in Human ES Cells Cells were transfected and data were analyzed as described in Figure 3B. Results from one of two independent experiments are shown. Bars represent average of duplicates performed in each single experiment, error bars depict standard deviation. Wild-type sequences, red bars; mutated sequences, blue bars.

Comparative DNA sequence analysis of pluripotency-associated genes revealed a high degree of conservation between mouse and human for several of the identified motifs (Figure 2C). To address whether the motifs active in mouse ES cells are also sufficient to activate transcription in human ES cells, we compared the expression levels of constructs bearing the identified motifs (Figure 5, red bars) with their mutated counterparts (blue bars). Interestingly, all of the motifs showed significant regulatory activity in human ES cells with levels similar to those in mouse ES cells. Likewise, the activity was diminished or abolished upon point mutations. These results underscore the power of our approach to predict and identify regulatory elements and suggest a strong degree of conservation in the transcriptional regulatory networks in mouse and human ES cells.

### Proteins Present in ES cells, Including the Transcription Factor NF-Y, Bind Sequence-Specifically to the Motifs

**Figure 6:**
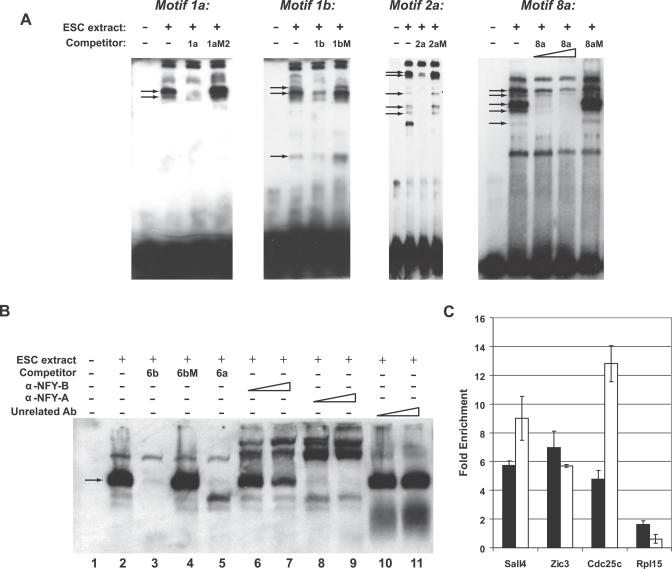
Proteins Present in ES Cells, Including NF-Y, Bind Sequence-Specifically to the Motifs (A) EMSA using motifs 1a, 1b, 2a and 8a. EMSA was performed with a double-stranded, biotin-labeled oligonucleotide containing the corresponding motif in the absence or presence of ES cell nuclear extracts. Where indicated, ES cell nuclear extracts were preincubated with a 200-fold molar excess of unlabeled competitor double-stranded oligonucleotides. For motif 8a, ES cell nuclear extracts were also preincubated with a 100-fold molar excess of unlabeled competitor double-stranded oligonucleotides. Arrows denote specific bands. (B) EMSA using motif 6. EMSA was performed with a double-stranded, biotin-labeled oligonucleotide containing motif 6b in the absence (lane 1) or presence (lanes 2–11) of ES cell nuclear extracts. ES cell nuclear extracts were preincubated with a 200-fold molar excess of unlabeled competitor double-stranded oligonucleotides (lanes 3–5), increasing amounts of α-NF-YB (lanes 6 and 7), α-NF-YA (lanes 8 and 9), or unrelated antibody (lanes 10 and 11). Arrow denotes the major specific band. (C) ChIP-real time PCR. Chromatin was precipitated from ES cell nuclear extracts using α-NF-YB or unrelated antibody. Data represent fold enrichment in the α-NF-YB precipitation relative to the unrelated antibody precipitation. *Cdc25c* is a known target of NF-Y [65] and *Sall4* and *Zic3* are two of the genes with the highest levels of upregulation in pluripotent cells (Figure 1A) that contain consensus NF-Y sites. *Rpl15* is a control gene that is not upregulated in ES cells. Black and white bars represent independent experiments performed with different ES cell nuclear extracts.

For several of the motifs, we performed electrophoretic mobility shift assays (EMSA) with biotin-labeled motif sequences. In the presence of ES cell nuclear protein extracts, shifted bands were observed for motifs 1a, 1b, 2a and 8a (Figure 6A). Several of these bands represent specific protein-motif complexes, as they were efficiently competed in the presence of excess unlabeled wild-type (1a, 1b, 2a and 8a, respectively) but not mutated probe (1aM, 1bM, 2aM and 8aM, respectively) (Figure 6A). These results show that proteins present in ES cells bind sequence-specifically to the motifs.

To identify putative transcription factors that can bind to the motifs, we systematically searched databases of known transcription factors. We did not find a match for most of the motifs, e.g., 1 and 2. We are particularly interested in identifying transcription factors that bind to these motifs, as they showed very high enhancer activity specifically in undifferentiated ES cells (Figure 3B; compare motifs 1a, 1b, 2a, and 2c to Oct4), but these transcription factors will have to be identified with unbiased biochemical or genetic approaches.

For motif 6 we were able to take a candidate factor approach. Two variants of the motif 6 (motifs 6a and 6b) contain a CCAAT box that when mutated caused a reduction in enhancer activity (Figures 4 and 5). CCAAT boxes have been shown to act as enhancers of transcription [61]. To identify the motif 6 binding factor(s), we performed EMSA. When biotin-labeled motif 6b was incubated with ES cell nuclear protein extracts, several shifted bands were observed (lane 2, Figure 6B). Excess unlabeled motif 6a and 6b, but not motif 6bM where the CCAAT box was mutated (Figure 4A), eliminated the binding of the major band, indicating that the band represents factor(s) specifically bound to the CCAAT box (lanes 3–5, Figure 6B). Several proteins able to bind CCAAT boxes have been described [62]. Among these is a heterotrimeric factor NF-Y (composed of NF-YA, NF-YB, and NF-YC subunits), which requires a high degree of conservation of the CCAAT sequence [63,64]. To determine whether NF-Y binds to the CCAAT box of motifs 6a and 6b, we performed additional EMSAs in which ES cell extracts were preincubated with anti-NF-Y antibodies (lanes 6–9, Figure 6B). The major motif 6b–protein complex was found to be specifically supershifted by antibodies against both NF-YA and NF-YB (lanes 6–9, Figure 6A), but not by an unrelated antibody (lanes 10 and 11, Figure 6B). These data show that NF-Y binds to motif 6, indicating that the NF-Y binding site is conserved and overrepresented in *cis*-acting regions of genes preferentially expressed in pluripotent cells.

To confirm that NF-Y binds directly to the promoters of genes upregulated in pluripotent cells, we performed chromatin immunoprecipitation (ChIP) real-time PCR. *Cdc25c* is a known target of NF-Y [65] and *Sall4* and *Zic3* are two of the genes with the highest levels of upregulation in pluripotent cells, present in the cluster in Figure 1A, that contain consensus NF-Y sites. Our ChIP data show that NF-Y binds to the CCAAT-containing regions of these genes in ES cells, but not to a control gene *(Rpl15)* that is not upregulated in ES cells (Figure 6C).

### NF-Y Is Differentially Expressed during ES Cell Differentiation

Even though NF-Y is expressed in several cell types and tissues [61], there is strong evidence for its differential expression: NF-YA and NF-YC are highly upregulated in mouse oocytes (40-fold and 12-fold, respectively, relative to the median expression in 60 other tissues) [66]. NF-YB was recently identified in a screen for genes upregulated in the inner cell mass of the mouse blastocyst [67]. In addition, alternative splicing produces two different NF-YA isoforms: NF-YA(long) and NF-YA(short) [68].

**Figure 7:**
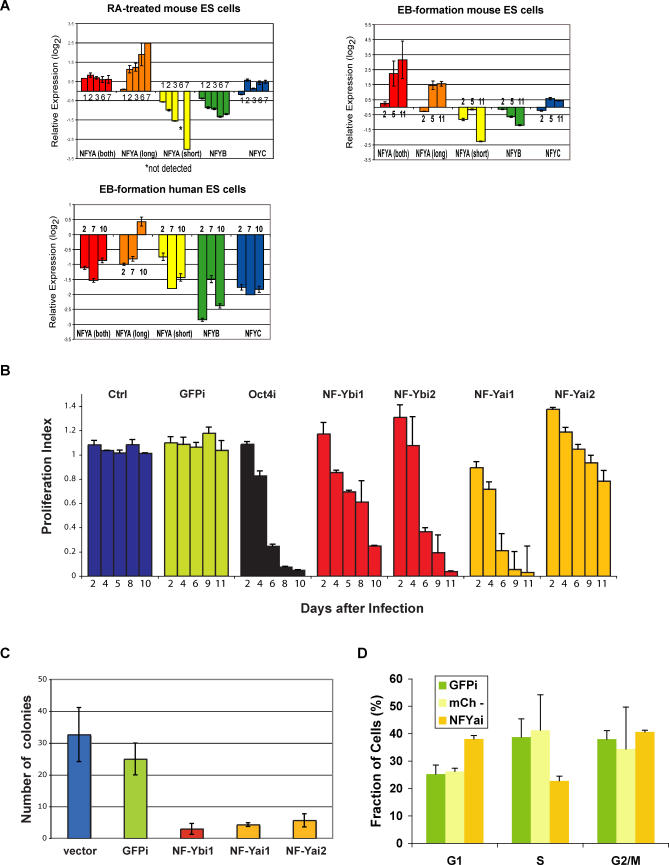
NF-Y Is Differentially Expressed during ES Cell Differentiation and Is Required for ES Cell Proliferation (A) Expression levels of NF-YA, NF-YA(long), NF-YA(short), NF-YB, and NF-YC during differentiation of ES cells. Real-time RT-PCR of RA-treated mouse ES cells, upper left panel; EB formation by mouse ES cells, upper right panel; EB formation by human ES cells, lower left panel. Fold-changes were calculated relative to undifferentiated ES cells using the REST software [94] and housekeeping genes as controls. A representative of at least three experiments (each performed in duplicate) is shown. Days of differentiation are indicated next to the bars. (B) RNAi-based competition assay. Mouse ES cells were infected with a lentiviral vector that induces RNAi and labels the cells with a red fluorescent marker, mCherry. The percentage of cells undergoing RNAi (mCherry+) was measured in a competition assay with noninfected wild-type cells over time. The ratio [mCherry+ cells in RNAi against target gene/mCherry+ cells in RNAi against GFP] gives a proliferation index. In the case of cells undergoing RNAi against GFP (GFPi), the ratio was calculated using cells infected with empty lentiviral vector as control. This index is expected to remain at 1 over time if there are no effects of RNAi against the target gene on proliferation and to be less than 1 if there are defects in cell proliferation. We validated our approach with RNAi against Oct4 (black bars). Downregulation of NF-YA or NF-YB leads to defective proliferation of ES cells (orange and red bars, respectively), while the unrelated control sequence or downregulation of GFP has no effect (blue and green bars, respectively). Results from one of 3–5 independent experiments are shown. Bars represent averages of duplicates (NF-YB, control) or triplicates (NF-YA, GFP, Oct4) performed in a single experiment. (C) Colony-formation assay. Control (vector and GFPi) cells and cells undergoing RNAi against NF-YA or B were sorted and plated at low density (300 cells per well of a 6-well plate) in triplicates. Colonies were counted after 10 d of culture. (D) Cell-cycle analysis. Cells infected with a lentivirus leading to NF-YA knockdown were sorted for mCherry+ cells (NF-YAi), which are undergoing RNAi against NF-YA, or mCherry- cells (mCh-), which correspond to in-plate control noninfected cells with which the NF-YA knockdown ES cells are competing. An additional control used was ES GFPi cells. Cells were stained using propidium iodide and analyzed for DNA content using flow cytometry. Samples were analyzed in triplicates. Error bars depict standard deviation.

We analyzed expression of NF-YA (including long and short isoforms), NF-YB, and NF-YC during mouse and human ES cell differentiation (Figure 7A). The levels of NF-Y mRNAs were analyzed by real-time reverse transcriptase PCR (RT-PCR) in mouse ES cells and differentiated ES cells either treated with RA or induced to form EBs. Interestingly, the expression of the two isoforms of NF-YA (long and short) changed in opposite directions; while the levels of NF-YA(long) increased with ES cell differentiation, NF-YA(short) was significantly downregulated with differentiation. At day 6 of RA-induced differentiation, NF-YA(short) was not detected, and at day 7 it was detected at low levels (8-fold reduction) (Figure 7A). The expression of NF-YB subunit was modestly reduced (up to 2.5-fold), while that of NF-YC did not change considerably during the course of differentiation. During differentiation of human ES cells, the expression of all NF-Y subunits was significantly reduced (Figure 7A). There is a lack of concordance of expression patterns for NF-YA(both), NF-YA(long), and NF-YC between the mouse and human EBs. It is possible that not all of the NF-Y subunits are regulated in identical manner in mouse and human ES cells, or that the cells forming in differentiated EBs, which are very heterogeneous cell populations, differ in nature or proportion in both species and have different levels of some NF-Y subunits (particularly NF-YA[long] and NF-YC). Nevertheless, our results show that NF-Y subunits, in particular NF-YA(short) and NF-YB, are downregulated during differentiation of mouse and human ES cells, suggesting that a specific subunit composition of NF-Y may be critical for ES cells.

### NF-Y Regulates ES Cell Proliferation

NF-Y has been implicated in promoting proliferation [69,70] and inhibiting differentiation [71] and senescence [72]. NF-YA mutant mice have been reported to display early embryonic lethality, as no mutant embryos were observed at the earliest stage analyzed (8.5 dpc) [70]. However, the function of NF-Y in ES cells had not been examined. We sought to investigate the role of NF-Y in proliferation of ES cells, using RNA interference (RNAi) in combination with a recently described competition assay [25]. This assay measures the ability of cells undergoing RNAi and grown in the presence of wild-type cells to maintain the rapid cell proliferation that characterizes wild-type ES cells (Figure 7B). The results are therefore a measure of the growth rate of ES cells undergoing RNAi relative to that of wild-type cells. We tested the effect of lentivirus-mediated NF-Y knockdown in mouse ES cells. As all three NF-Y subunits are required for sequence-specific DNA binding and downregulation of any subunit is expected to impair NF-Y binding to DNA [63,64], we infected GFP-expressing ES cells [73] with either NF-YA or NF-YB short hairpin RNAs (shRNAs). Similar to the control Oct4 shRNA [25], cells infected with NF-YA or NF-YB shRNAs were selectively out-competed by wild-type cells over time (NF-Yai1 and NF-Ybi1, Figure 7B). To confirm the RNAi specificity and exclude possible off-target effects, we tested NF-YA and NF-YB shRNAs that target a different region of the mRNAs (NF-Yai2 and NF-Ybi2, respectively, Figure 7B). In addition, an unrelated shRNA and an shRNA targeting GFP transcript were used to exclude the possibility that the effects observed were due to sequestration of the RNAi machinery, rather than depletion of specific gene products (ctrl and GFPi, respectively, Figure 7B). While the percentage of cells infected with the unrelated or GFP shRNAs did not change significantly, cells infected with the NF-Yai2 and NF-Ybi2 shRNAs were out-competed by noninfected, wild-type cells. The specificity of the NF-YA and NF-YB knockdown was confirmed by real-time RT-PCR (unpublished data). Our preliminary results using real-time RT-PCR for several differentiation markers do not reveal induction of differentiation upon RNAi. This suggests that the primary role of NF-Y may be to maintain the high proliferative capacity of ES cells.

We characterized the role of NF-Y in ES cell proliferation in more detail. Plating cells at low density revealed a strong decrease in the clonogenic potential of ES cells undergoing RNAi against NF-YA or B, relative to control cells (Figure 7C). Using staining for alkaline phosphatase, a marker of undifferentiated ES cell colonies, we did not observe partially stained or unstained colonies upon NF-Y knockdown. The very few colonies that formed were still alkaline phosphatase positive, and may be due to less than 100% pure FACS isolation of cells undergoing RNAi prior to plating, or to incomplete knockdown of NF-Y to levels that still allow colony formation. These data indicate that knockdown of NF-YA or NF-YB compromises the clonogenic potential of mouse ES cells, reducing it by 5–10-fold. Cell cycle analysis using NF-YA knockdown ES cells revealed an increased proportion of cells in G1 and a decreased proportion of cells in S phase (Figure 7D). Taken together, these results indicate that inhibition of NF-Y function leads to defects in ES cell proliferation that correlates with an accumulation of cells at the G1/S transition of the cell cycle.

## Discussion

In this study we report a systematic approach that combines comprehensive expression analysis of coregulated genes, computational de novo motif prediction, biochemical validation of *cis*-regulatory elements, and identification of transcription factors that bind to those elements in pluripotent stem cells. Our methodology can be used with any set of coregulated genes, and, as such, is broadly applicable to the characterization of transcriptional regulatory networks. The approach we describe compares favorably to the standard experimental method to identify regulatory sequences, which relies on time-consuming dissection of large noncoding regions of a single gene. When compared to other methods to identify *cis*-regulatory elements, like ChIP in combination with microarrays (ChIP-chip) or paired-end ditag sequencing (ChIP-PET), our approach has two principal advantages: it does not require prior knowledge of the critical transcription factors whose targets are to be investigated, and it is not limited by the number of cells available for analysis. In particular, we have been able to generate reliable expression data from as low as 500–1,000 cells (unpublished data), whereas current ChIP-chip and ChIP-PET methods require several million cells [23,74,75]. Thus, we envision that the approach described here will be particularly useful for the characterization of transcriptional networks that regulate cell fate decisions during embryonic development and stem cell differentiation.

We identified short DNA sequence motifs that are highly active in undifferentiated ES cells but not in differentiated cells (Figure 3B, motifs 1 and 2). Importantly, the level of activity of these motifs is significantly higher than that of the Oct4/Sox2 element in the Nanog promoter (Figure 3B, compare motifs 1a, 1b, 2a, and 2c to Nng). These results indicate that we identified enhancer elements that are bound by transcriptional factors preferentially active in undifferentiated mouse and human ES cells. The availability of EMSAs for motifs 1 and 2 and of mutated versions that highly reduce or abolish motif activity (Figures 4, 5, and 6A) should facilitate the unbiased identification of the transcription factors that bind to these motifs.

An important validation of our systematic analysis of *cis*-regulatory elements active in ES cells is the identification of NF-Y as a transcription factor that binds specifically to one of those elements and regulates ES cell proliferation. In support of our findings, the NF-Y binding site was detected as overrepresented in genomic regions bound by Oct4 and Sox2 in human ES cells (Qing Zhou and Wing Wong, personal communication). It is possible that NF-Y contributes to the regulation of the peculiar cell cycle pattern of ES cells, with a short G1 phase and insensitivity to the Rb pathway (reviewed in [76]). NF-Y had previously been shown to regulate cell proliferation in other experimental paradigms [69,70], but its role in early embryonic development remains poorly understood. The strong upregulation of subunits of NF-Y in oocytes [66] and the ICM [67], and the early arrest of NF-YA mutant embryos [70], indicates that NF-Y plays important roles during early embryogenesis. It is also worth noting the dramatic difference in expression of NF-YA isoforms during ES cell differentiation (Figure 7A). Both NF-YA isoforms contain a glutamine-rich region that is reduced in the short isoform of NF-YA [68]. The glutamine-rich region of NF-YA has been shown to activate transcription [68,77,78] and it is also a protein–protein interaction domain [79]. The functional significance of the two NF-YA isoforms remains to be elucidated, although recent data indicate that NF-YA(short) promotes self-renewal of hematopoietic stem cells [80]. Future studies will address the specific contribution of NF-Y and its different subunits, in particular NF-YA(short), in ES cells.

ES cells may be governed at the molecular level by the action of cell-specific transcription factors, such as Oct4 and Nanog, and factors that are also expressed in other cell types, such as NF-Y, c-Myc [50], and Stat3 [11]. Interestingly, NF-Y binds to the promoter of Sall4 (Figure 6C), an essential ES cell regulator [24]. It will be important to identify the target genes that are regulated by NF-Y in ES cells. We expect that the combination of ChIP-chip and expression profiling will reveal the contribution of NF-Y to the transcriptional program of ES cells.

In summary, we report here the identification of clusters of genes upregulated in pluripotent cells, the development of a novel algorithm for discovery of short *cis*-acting regulatory motifs, the validation of the activity of several novel motifs in mouse and human pluripotent stem cells, and the identification of transcription factor NF-Y as a regulator of gene expression in ES cells that is required for their proliferation. Genetic and biochemical approaches should allow the identification of other transcription factors that bind to the motifs. Our results provide a basis for understanding the transcriptional regulatory networks that underlie early mammalian embryogenesis and ES cell self-renewal and pluripotency.

## Materials and Methods

### Microarray data analysis.

The isolation of PGCs and SGM cells from 11.5 dpc Oct4/GFP transgenic mouse embryos and the identification of their transcription profiles is described elsewhere (Wei et al, submitted). Briefly, we used 20,000–30,000 PGCs or SGM cells per replicate sample, and analyzed 3–4 replicates per tissue using Affymetrix U74Av2 arrays (http://www.affymetrix.com), which assay for the expression of about 12,000 genes. We normalized, modeled, and clustered gene expression profiles (Dataset S1) using the dChip software (http://biosun1.harvard.edu/complab/dchip/) [81]. We compared the gene expression profiles of PGCs and SGM cells with those of embryonic and adult stem cells that we had previously described [42]. Hierarchical clustering was used to identify clusters of genes associated with pluripotency. A total of 230 probe sets were selected and used for Gene Ontology term analyses with the Onto-Express software (http://vortex.cs.wayne.edu/) [43]. *p*-Values for significance of overrepresentation of functional annotations were calculated in Onto-Express using a hypergeometric distribution and corrected for multiple testing using false discovery rate. For motif discovery, a cluster of 55 probe sets (included in the 230 probe sets used for Gene Ontology term analyses) was selected by the additional criteria: downregulation in differentiation of ES cells towards EBs (H. Chipperfield, S. Zhong, D. Melton, and W. Wong, personal communication); standard deviation/mean > 0.6. These 55 probe sets are listed in Dataset S7. Detailed protocols are available upon request.

### Computational methods.

To identify putative motifs shared among the pluripotency-associated gene cluster, Affymetrix probe sets were mapped to Ensembl (http://www.ensembl.org) gene annotation v.27 (Dataset S17) [82,83]. Both 1,000 bp and 2,000 bp of the intergenic sequences upstream from the transcriptional start site were extracted for each gene. For each of the different lengths, three sets of sequences were extracted from Ensembl; the first set contained the sequences of all annotated genes in the cluster in mouse (build 33), the second set contained the sequences from the orthologous human (build 35) genes in the cluster obtained from Ensembl mapping [83,84], and the last set consisted only of concatenated blocks of mouse promoter sequences that were conserved between mouse and human within the specified upstream sequence length. Pairwise alignments between mouse (mm5) and human (hg17) were obtained from the University of California Santa Cruz Genome Browser database (http://genome.ucsc.edu) [85–87].

Next, the three sets of upstream sequences were repeat masked (http://www.repeatmasker.org) and then used as input for the MobyDick algorithm [28,88] to build three dictionaries of putative motifs. The adjustable parameters used were as described [57], except MaxP, which was set to 0.1. Similar motifs from all dictionaries were grouped into one final dictionary of motif clusters using the CAST clustering algorithm [89]. All pairs of motifs in the dictionaries were scored based on a gapless pairwise alignment using a simple mutation model [57], after which CAST was applied with the threshold parameter set at 0.55 (the lower bound of the normalized score averaged over all pairs in a cluster).

Following the clustering step, we calculated a *p*-value to identify motif clusters that were significantly overrepresented in the pluripotency-associated gene cluster compared to a background contrast set. As our contrast set, we used about 8,500 mouse promoter regions from the genes on the Affymetrix U74Av2 platform not in the pluripotency-associated gene cluster. To calculate the *p*-values, we counted the number of occurrences of each motif cluster in the contrast set and calculated the expected number of occurrences based on a random distribution throughout the genes, *N*_exp_. We then counted the number of occurrences of each motif cluster within the mouse promoter regions of the pluripotency-associated gene cluster, *N*_obs_. Poisson distribution was then used to calculate the probability *p* of observing the number of occurrences equal to or greater than *N*_obs_ by chance given the expected number of occurrences *N*_exp_. These *p*-values were Bonferroni corrected by the number of clusters.

Ten motif clusters (Table S1) were selected for further experimental characterization based on the following criteria: −log_10_ *p*-values greater than zero after correction for multiple testing, evolutionary conservation across different promoter dictionaries, copy number less than 50, and nonrepetitive elements. From our list of ten motif clusters, we identified highly conserved motifs by searching for all occurrences of each motif within the mouse sequence of the alignment between human, mouse, rat, and dog [44] and extended the motif to the flanking regions if the flanking regions were also highly conserved (Dataset S16). A highly conserved position was defined to be a nucleotide base that was conserved across all four species.

### Construction of reporter vectors.

The 242-bp fragment of *Oct4* DE (−2,181 to −1,939) was PCR amplified from GOF18ΔPE/EGFP plasmid [90] using primers Oct4_2 and Oct4_3 (Dataset S18) containing BglII and BamHI restriction sites. The PCR product was digested and cloned into the BglII/BamHI digested plasmid pFoXLucTK [91]. Nng and motif-containing plasmids were cloned by hybridizing complementary oligos (Dataset S18) that yielded BglII and BamHI restriction site overhangs and ligating them to BglII/BamHI-digested plasmid pFoXTKLuc. Upstream genomic sequences of *Eed* were PCR amplified from the mouse genome with primers Eed2 and Eed4 (Dataset S18) and cloned into the pCRII-TOPO vector (Invitrogen, http://www.invitrogen.com), from which it was subsequently excised by SpeI/XbaI restriction digest and ligated to SpeI/XbaI digested pFoxLuc vector [91]. All plasmids were verified by sequencing.

### Cell culture and differentiation.

Mouse E14 ES and EG cells were maintained in Dulbecco's modified Eagle's medium (DMEM) (Invitrogen) supplemented with 10% fetal bovine serum, 1mM L-glutamine, 0.1 mM nonessential amino acids, 100u/mL penicillin, 100 μg/ml streptomycin, 1 mM sodium pyruvate, 0.1 mM 2-mercaptoethanol, and recombinant LIF. Mouse Oct4/GFP ES cells [73] were grown in identical conditions except that knockout serum replacement (Invitrogen) was used instead of fetal bovine serum. Human ES H9 cells were cultured in Knockout DMEM supplemented with 20% knockout serum replacement (Invitrogen), 1mM L-glutamine, 0.1 mM nonessential amino acids, 0.1 mM 2-mercaptoethanol, and 10 ng/ml recombinant human FGF-2 on X-ray inactivated mouse embryonic fibroblasts. Embryoid bodies were formed by suspension culturing, and chemical differentiation induction was performed with 0.5 μM all-trans-RA (Sigma, http://www.sigmaaldrich.com/), both in the absence of LIF. HEK293 and NIH-3T3 cells were cultured in DMEM containing 10% fetal bovine serum, 1mM L-glutamine, 100u/ml penicillin, 100 μg/ml streptomycin, and 0.1 mM nonessential amino acids.

### Transfection and luciferase reporter assays.

1.5 × 10^5^ cells were plated in 12-well tissue culture plates 24 h prior to transfection. Human ES cells were plated on Matrigel (BD Bioscience, http://www.bdbiosciences.com/), in the absence of mouse feeder cells. Each reporter construct (500 ng) was cotransfected with the pRL-TK vector (200 ng) (Promega http://www.promega.com/) as an internal control using 2 μl of Lipofectamine2000 (Invitrogen), according to manufacturer's instructions. Cells were lysed 24 h after transfection, and luciferase activities were measured using a dual-luciferase assay system (Promega).

### Electrophoretic mobility-shift assay.

Biotin-labeled double-stranded oligos containing motif 6a and 6b sequences (Figure 4A) were incubated with 10 μg mouse ES cell nuclear extracts using the LightShift Chemiluminescent EMSA Kit (Pierce, http://www.piercenet.com/). The formation of DNA–protein complexes was analyzed by 5% nondenaturing polyacrylamide gel electrophoresis, followed by semi-dry transfer to the GeneScreen membrane (PerkinElmer, http://www.perkinelmer.com/) and biotin detection using the LightShift Kit according to manufacturer's instructions. For supershift assays, 2 μg or 6 μg of α-NF-YA (ab6558; Abcam, http://www.abcam.com/) or α-NF-YB (ab6559, Abcam) were added.

### ChIP.

ChIP was performed essentially as described in [23] and by Upstate Biotechnology (http://www.upstate.com). Briefly, chromatin was cross-linked using 1% formaldehyde for 10 min, the reaction was quenched with 1/20 volume of 2.5 M glycine and centrifuged at 1,350 ×g for 5 min, and the pellet was washed with PBS and sonicated to obtain fragments of ∼100–600 bp, as verified on a gel. Reactions were centrifuged at 20,000 ×g for 10 min and the supernatants were used for incubations with α-NF-YB (FL207; Santa Cruz Biotechnology, http://www.scbt.com/) or α-V5 (ab9137, Abcam) overnight at 4 °C. Dynal Protein G beads (Invitrogen) were used for magnetic recovery of antibody-bound chromatin following the manufacturer's instructions. Crosslinking was reversed by incubation at 65 °C overnight. Reactions were digested with RNAse A and Proteinase K and DNA was purified by phenol–chloroform extraction and ethanol precipitation. DNA concentration was determined using a Nanodrop (NanoDrop Technologies, http://www.nanodrop.com/) and 8 ng were used in Sybr Green real-time PCRs (see below) ran in duplicates or triplicates. Primer sequences are available upon request. Fold enrichment was calculated using the 2^ΔCt^ method. The gene *Rpl15* was used as control. All PCRs were verified on a gel for the presence of a single band of the correct size.

### RNAi and competition assay.

shRNA sequences were selected according to published criteria [92]: GFPi-ACAGCCACAACGTCTATAT, Oct4i-GAACCTGGCTAAGCTTCCA, NF-YBi1-GTAGTTCTAGCTCTATCAA, NF-YBi2-GACTAATTGAGGTGTTAAT, NF-YAi1-GAGACAGTTTAGAGAGTAA, NF-YAi2- GAAGTGTTGAGGACATTCA, and control-ACAGCCACAACGTCTATAT. Oligos coding for the shRNAs were designed and cloned into the lentiviral vector pSicoR-mCherry as described [93]. pSicoR-mCherry was generated by replacing mCherry for GFP in pSicoR.

Lentiviruses were produced as described [93]. For transduction, 10^6^ ES cells were incubated with virus in 1 ml of ES cell medium (multiplicity of infection 5–10). After 1 h rotating at 37 °C, 2.5 × 10^5^–3 × 10^5^ cells were plated per gelatinized well of a 12-well plate. Cells were passaged and a sample collected for analysis every 2 d. The percentage of mCherry+ cells was determined and mCherry+ and mCherry− cells were isolated using a FACSDiVa (BD Biosciences) cell sorter.

### Real-time RT-PCR.

RNA was isolated and reverse transcribed using the iScript first strand cDNA synthesis kit (Bio-Rad Laboratories, http://www.bio-rad.com/). The cDNA reaction was diluted 1:5 in TE and used in Sybr Green real-time PCRs (Bio-Rad Laboratories). PCR primers were designed to amplify 100–200-bp fragments spanning two exons at the 3′ end of the gene. Housekeeping genes used were *Ppia* (for mouse), *Ubb,* and *ribosomal protein L7* (for mouse and human), which were determined from the microarray data to not be differentially expressed in the samples analyzed. PCR primer sequences are available upon request. Reactions were run in replicates on a MyiQ qPCR machine (Bio-Rad Laboratories) according to the manufacturer's instructions. Only samples with single and matching end-point melting curve peaks were used for subsequent analysis. Cycle threshold values were imported into the REST software [94] for fold-change calculations, using the housekeeping genes as controls.

### Colony formation assay.

Cells were infected with lentiviruses containing shRNAs and mCherry, as described above. mCherry+ and mCherry− cells were isolated using a FACSDiVa (BD Biosciences) cell sorter. Three hundred cells were plated per well of a 6-well plate in triplicates. After 10 d in culture, cells were stained for alkaline phosphatase using a Vector kit (http://www.vectorlabs.com/) and colonies were counted.

### Cell cycle analysis.

Cells were infected with lentiviruses leading to the expression of shRNAs and mCherry, as described above. mCherry+ and mCherry− cells were isolated using a FACSDiVa (BD Biosciences) cell sorter. Cells were washed twice with cold PBS, resuspended at concentration of 2 × 10^6^ cells/ml in PBS and fixed with cold ethanol. After overnight incubation at 4 °C, cells were washed twice and resuspended in 160 μl PBS containing 1% BSA. Twenty microliters of propidium iodide (0.5 mg/ml) and 20 μl of RNase A (10 mg/ml) were added, cells were incubated at 37 °C for 30 min, and analysis was performed using a FACScalibur flow cytometer and FloJo.

## Supporting Information

Dataset S1 Normalized Microarray Expression Data (6.3 MB XLS)

Dataset S2 Genes in Microarray Expression Cluster from Figure 1A (96 KB XLS)

Dataset S3 Gene Ontology Analysis of All Categories (785 KB XLS)

Dataset S4 Gene Ontology Analysis of Cellular Compartment (61 KB XLS)

Dataset S5 Gene Ontology Analysis of Biological Process (100 KB XLS)

Dataset S6 Gene Ontology Analysis of Molecular Function (88 KB XLS)

Dataset S7 Affymetrix Probe Sets Used in CompMoby Analysis (30 KB XLS)

Dataset S8 CompMoby Results for 1-kb Mouse Dictionary (41 KB XLS)

Dataset S9 CompMoby Results for 1-kb Human Dictionary (48 KB XLS)

Dataset S10 CompMoby Results for 1-kb Conserved Blocks Dictionary (42 KB XLS)

Dataset S11 CompMoby Results for 2-kb Mouse Dictionary (47 KB XLS)

Dataset S12 CompMoby Results for 2-kb Human Dictionary (42 KB XLS)

Dataset S13 CompMoby Results for 2-kb Conserved Blocks Dictionary (46 KB XLS)

Dataset S14 CompMoby Results for 1-kb Final Dictionary Where All 1-kb Dictionaries Are Clustered by Sequence Similarity (59 KB XLS)

Dataset S15 CompMoby Results for 2-kb Final Dictionary Where All 2-kb Dictionaries Are Clustered by Sequence Similarity (40 KB XLS)

Dataset S16 Conservation of Motif Clusters across Four Species Alignment of Human, Mouse, Rat, and Dog (27 KB PDF)

Dataset S17 Ensembl Probes Used for CompMoby Analysis (14 KB XLS)

Dataset S18 Oligos Used for Motifs (22 KB XLS)

Figure S1 Increase in the Number of Motif Repeats Leads to a Proportional Increase in Motif Activity Predicted motif sequences 1b, 2c and 8a were fused to a construct containing Firefly luciferase driven by a minimal TK promoter. The number of repeats for a given motif is indicated below corresponding bar graph. Constructs 2c and 8a bear one or two repeats of genomic sequence that each contain two identical predicted regulatory motifs. The constructs were transfected in undifferentiated mouse ES cells. A plasmid containing Renilla luciferase driven by the TK promoter was cotransfected. After 24 h, cells were lysed and assayed for luciferase activities. Firefly values were corrected for Renilla values, and the activities of the tested constructs were calculated relative to the activity of the TK construct, which was set to 1. Representative results from two to five independent experiments are shown. Bars represent averages of triplicates performed in each single experiment. Error bars depict standard deviation.
(220 KB PDF)

Table S1 Position of Motifs from Transcriptional Start Sites (40 KB XLS)

### Accession Numbers

The gene expression profiles of ES cells, PGCs, and somatic cells can be obtained from ArrayExpress (http://www.ebi.ac.uk/arrayexpress/), accession number E-MEXP-1158.

## Abbreviations

ChIP: chromatin immunoprecipitation
dpc: days post coitum
EB: embryoid body
EG: embryonic germ
ES: embryonic stem
LIF: leukemia inhibitory factor
PGC: primordial germ cell
RA: retinoic acid
RNAi: RNA interference
RT-PCR: reverse transcriptase PCR
SGM: somatic cells of the genital ridge/mesonephros area
shRNA: short hairpin RNA
TK: thymidine kinase

## References

[pgen-0030145-b001] Evans MJ, Kaufman MH (1981). Establishment in culture of pluripotential cells from mouse embryos. Nature.

[pgen-0030145-b002] Martin GR (1981). Isolation of a pluripotent cell line from early mouse embryos cultured in medium conditioned by teratocarcinoma stem cells. Proc Natl Acad Sci U S A.

[pgen-0030145-b003] Pera MF, Trounson AO (2004). Human embryonic stem cells: Prospects for development. Development.

[pgen-0030145-b004] Donovan PJ, de Miguel MP (2003). Turning germ cells into stem cells. Curr Opin Genet Dev.

[pgen-0030145-b005] Matsui Y, Okamura D (2005). Mechanisms of germ-cell specification in mouse embryos. Bioessays.

[pgen-0030145-b006] Matsui Y, Zsebo K, Hogan BL (1992). Derivation of pluripotential embryonic stem cells from murine primordial germ cells in culture. Cell.

[pgen-0030145-b007] Resnick JL, Bixler LS, Cheng L, Donovan PJ (1992). Long-term proliferation of mouse primordial germ cells in culture. Nature.

[pgen-0030145-b008] Boiani M, Scholer HR (2005). Regulatory networks in embryo-derived pluripotent stem cells. Nat Rev Mol Cell Biol.

[pgen-0030145-b009] Smith AG, Heath JK, Donaldson DD, Wong GG, Moreau J (1988). Inhibition of pluripotential embryonic stem cell differentiation by purified polypeptides. Nature.

[pgen-0030145-b010] Williams RL, Hilton DJ, Pease S, Willson TA, Stewart CL (1988). Myeloid leukaemia inhibitory factor maintains the developmental potential of embryonic stem cells. Nature.

[pgen-0030145-b011] Niwa H, Burdon T, Chambers I, Smith A (1998). Self-renewal of pluripotent embryonic stem cells is mediated via activation of STAT3. Genes Dev.

[pgen-0030145-b012] Ying QL, Nichols J, Chambers I, Smith A (2003). BMP induction of Id proteins suppresses differentiation and sustains embryonic stem cell self-renewal in collaboration with STAT3. Cell.

[pgen-0030145-b013] Mitsui K, Tokuzawa Y, Itoh H, Segawa K, Murakami M (2003). The homeoprotein Nanog is required for maintenance of pluripotency in mouse epiblast and ES cells. Cell.

[pgen-0030145-b014] Chambers I, Colby D, Robertson M, Nichols J, Lee S (2003). Functional expression cloning of Nanog, a pluripotency sustaining factor in embryonic stem cells. Cell.

[pgen-0030145-b015] Nichols J, Zevnik B, Anastassiadis K, Niwa H, Klewe-Nebenius D (1998). Formation of pluripotent stem cells in the mammalian embryo depends on the POU transcription factor Oct4. Cell.

[pgen-0030145-b016] Niwa H, Miyazaki J, Smith AG (2000). Quantitative expression of Oct-3/4 defines differentiation, dedifferentiation or self-renewal of ES cells. Nat Genet.

[pgen-0030145-b017] Reubinoff BE, Pera MF, Fong CY, Trounson A, Bongso A (2000). Embryonic stem cell lines from human blastocysts: Somatic differentiation in vitro. Nat Biotechnol.

[pgen-0030145-b018] Thomson JA, Itskovitz-Eldor J, Shapiro SS, Waknitz MA, Swiergiel JJ (1998). Embryonic stem cell lines derived from human blastocysts. Science.

[pgen-0030145-b019] Pera MF, Andrade J, Houssami S, Reubinoff B, Trounson A (2004). Regulation of human embryonic stem cell differentiation by BMP-2 and its antagonist noggin. J Cell Sci.

[pgen-0030145-b020] Xu RH, Chen X, Li DS, Li R, Addicks GC (2002). BMP4 initiates human embryonic stem cell differentiation to trophoblast. Nat Biotechnol.

[pgen-0030145-b021] Hay DC, Sutherland L, Clark J, Burdon T (2004). Oct-4 knockdown induces similar patterns of endoderm and trophoblast differentiation markers in human and mouse embryonic stem cells. Stem Cells.

[pgen-0030145-b022] Zaehres H, Lensch MW, Daheron L, Stewart SA, Itskovitz-Eldor J (2005). High-efficiency RNA interference in human embryonic stem cells. Stem Cells.

[pgen-0030145-b023] Boyer LA, Lee TI, Cole MF, Johnstone SE, Levine SS (2005). Core transcriptional regulatory circuitry in human embryonic stem cells. Cell.

[pgen-0030145-b024] Zhang J, Tam WL, Tong GQ, Wu Q, Chan HY (2006). Sall4 modulates embryonic stem cell pluripotency and early embryonic development by the transcriptional regulation of Pou5f1. Nat Cell Biol.

[pgen-0030145-b025] Ivanova N, Dobrin R, Lu R, Kotenko I, Levorse J (2006). Dissecting self-renewal in stem cells with RNA interference. Nature.

[pgen-0030145-b026] Pennacchio LA, Rubin EM (2001). Genomic strategies to identify mammalian regulatory sequences. Nat Rev Genet.

[pgen-0030145-b027] MacIsaac KD, Fraenkel E (2006). Practical strategies for discovering regulatory DNA sequence motifs. PLoS Comput Biol.

[pgen-0030145-b028] Bussemaker HJ, Li H, Siggia ED (2000). Building a dictionary for genomes: Identification of presumptive regulatory sites by statistical analysis. Proc Natl Acad Sci U S A.

[pgen-0030145-b029] Hertz GZ, Stormo GD (1999). Identifying DNA and protein patterns with statistically significant alignments of multiple sequences. Bioinformatics.

[pgen-0030145-b030] Lawrence CE, Altschul SF, Boguski MS, Liu JS, Neuwald AF (1993). Detecting subtle sequence signals: A Gibbs sampling strategy for multiple alignment. Science.

[pgen-0030145-b031] Liu X, Brutlag DL, Liu JS (2001). BioProspector: Discovering conserved DNA motifs in upstream regulatory regions of co-expressed genes. Pac Symp Biocomput.

[pgen-0030145-b032] Bailey TL, Elkan C (1995). The value of prior knowledge in discovering motifs with MEME. Proc Int Conf Intell Syst Mol Biol.

[pgen-0030145-b033] Tompa M, Li N, Bailey TL, Church GM, De Moor B (2005). Assessing computational tools for the discovery of transcription factor binding sites. Nat Biotechnol.

[pgen-0030145-b034] GuhaThakurta D (2006). Computational identification of transcriptional regulatory elements in DNA sequence. Nucleic Acids Res.

[pgen-0030145-b035] Mootha VK, Handschin C, Arlow D, Xie X, St Pierre J (2004). Erralpha and Gabpa/b specify PGC-1alpha-dependent oxidative phosphorylation gene expression that is altered in diabetic muscle. Proc Natl Acad Sci U S A.

[pgen-0030145-b036] Wang T, Stormo GD (2003). Combining phylogenetic data with co-regulated genes to identify regulatory motifs. Bioinformatics.

[pgen-0030145-b037] Liu Y, Liu XS, Wei L, Altman RB, Batzoglou S (2004). Eukaryotic regulatory element conservation analysis and identification using comparative genomics. Genome Res.

[pgen-0030145-b038] Siddharthan R, Siggia ED, van Nimwegen E (2005). PhyloGibbs: A Gibbs sampling motif finder that incorporates phylogeny. PLoS Comput Biol.

[pgen-0030145-b039] Emberly E, Rajewsky N, Siggia ED (2003). Conservation of regulatory elements between two species of Drosophila. BMC Bioinformatics.

[pgen-0030145-b040] Dermitzakis ET, Clark AG (2002). Evolution of transcription factor binding sites in Mammalian gene regulatory regions: Conservation and turnover. Mol Biol Evol.

[pgen-0030145-b041] Yoshimizu T, Sugiyama N, De Felice M, Yeom YI, Ohbo K (1999). Germline-specific expression of the Oct-4/green fluorescent protein (GFP) transgene in mice. Dev Growth Differ.

[pgen-0030145-b042] Ramalho-Santos M, Yoon S, Matsuzaki Y, Mulligan RC, Melton DA (2002). “Stemness”: transcriptional profiling of embryonic and adult stem cells. Science.

[pgen-0030145-b043] Khatri P, Draghici S, Ostermeier GC, Krawetz SA (2002). Profiling gene expression using onto-express. Genomics.

[pgen-0030145-b044] Xie X, Lu J, Kulbokas EJ, Golub TR, Mootha V (2005). Systematic discovery of regulatory motifs in human promoters and 3′ UTRs by comparison of several mammals. Nature.

[pgen-0030145-b045] Kuroda T, Tada M, Kubota H, Kimura H, Hatano SY (2005). Octamer and Sox elements are required for transcriptional *cis* regulation of Nanog gene expression. Mol Cell Biol.

[pgen-0030145-b046] Rodda DJ, Chew JL, Lim LH, Loh YH, Wang B (2005). Transcriptional regulation of nanog by OCT4 and SOX2. J Biol Chem.

[pgen-0030145-b047] Blackwell TK, Huang J, Ma A, Kretzner L, Alt FW (1993). Binding of myc proteins to canonical and noncanonical DNA sequences. Mol Cell Biol.

[pgen-0030145-b048] Blackwell TK, Kretzner L, Blackwood EM, Eisenman RN, Weintraub H (1990). Sequence-specific DNA binding by the c-Myc protein. Science.

[pgen-0030145-b049] Massari ME, Murre C (2000). Helix-loop-helix proteins: Regulators of transcription in eucaryotic organisms. Mol Cell Biol.

[pgen-0030145-b050] Cartwright P, McLean C, Sheppard A, Rivett D, Jones K (2005). LIF/STAT3 controls ES cell self-renewal and pluripotency by a Myc-dependent mechanism. Development.

[pgen-0030145-b051] Takahashi K, Yamanaka S (2006). Induction of pluripotent stem cells from mouse embryonic and adult fibroblast cultures by defined factors. Cell.

[pgen-0030145-b052] Yuan H, Corbi N, Basilico C, Dailey L (1995). Developmental-specific activity of the FGF-4 enhancer requires the synergistic action of Sox2 and Oct-3. Genes Dev.

[pgen-0030145-b053] Tomioka M, Nishimoto M, Miyagi S, Katayanagi T, Fukui N (2002). Identification of Sox-2 regulatory region which is under the control of Oct-3/4-Sox-2 complex. Nucleic Acids Res.

[pgen-0030145-b054] Tokuzawa Y, Kaiho E, Maruyama M, Takahashi K, Mitsui K (2003). Fbx15 is a novel target of Oct3/4 but is dispensable for embryonic stem cell self-renewal and mouse development. Mol Cell Biol.

[pgen-0030145-b055] Nishimoto M, Fukushima A, Okuda A, Muramatsu M (1999). The gene for the embryonic stem cell coactivator UTF1 carries a regulatory element which selectively interacts with a complex composed of Oct-3/4 and Sox-2. Mol Cell Biol.

[pgen-0030145-b056] Okumura-Nakanishi S, Saito M, Niwa H, Ishikawa F (2005). Oct-3/4 and Sox2 regulate Oct-3/4 gene in embryonic stem cells. J Biol Chem.

[pgen-0030145-b057] Patil CK, Li H, Walter P (2004). Gcn4p and novel upstream activating sequences regulate targets of the unfolded protein response. PLoS Biol.

[pgen-0030145-b058] Perini G, Diolaiti D, Porro A, Della Valle G (2005). In vivo transcriptional regulation of N-Myc target genes is controlled by E-box methylation. Proc Natl Acad Sci U S A.

[pgen-0030145-b059] Safe S, Abdelrahim M (2005). Sp transcription factor family and its role in cancer. Eur J Cancer.

[pgen-0030145-b060] Boyer LA, Plath K, Zeitlinger J, Brambrink T, Medeiros LA (2006). Polycomb complexes repress developmental regulators in murine embryonic stem cells. Nature.

[pgen-0030145-b061] Mantovani R (1999). The molecular biology of the CCAAT-binding factor NF-Y. Gene.

[pgen-0030145-b062] Matuoka K, Chen KY (2002). Transcriptional regulation of cellular ageing by the CCAAT box-binding factor CBF/NF-Y. Ageing Res Rev.

[pgen-0030145-b063] Maity SN, Sinha S, Ruteshouser EC, de Crombrugghe B (1992). Three different polypeptides are necessary for DNA binding of the mammalian heteromeric CCAAT binding factor. J Biol Chem.

[pgen-0030145-b064] Sinha S, Maity SN, Lu J, de Crombrugghe B (1995). Recombinant rat CBF-C, the third subunit of CBF/NFY, allows formation of a protein-DNA complex with CBF-A and CBF-B and with yeast HAP2 and HAP3. Proc Natl Acad Sci U S A.

[pgen-0030145-b065] Zwicker J, Gross C, Lucibello FC, Truss M, Ehlert F (1995). Cell cycle regulation of cdc25C transcription is mediated by the periodic repression of the glutamine-rich activators NF-Y and Sp1. Nucleic Acids Res.

[pgen-0030145-b066] Su AI, Wiltshire T, Batalov S, Lapp H, Ching KA (2004). A gene atlas of the mouse and human protein-encoding transcriptomes. Proc Natl Acad Sci U S A.

[pgen-0030145-b067] Yoshikawa T, Piao Y, Zhong J, Matoba R, Carter MG (2006). High-throughput screen for genes predominantly expressed in the ICM of mouse blastocysts by whole mount in situ hybridization. Gene Expr Patterns.

[pgen-0030145-b068] Li XY, Hooft van Huijsduijnen R, Mantovani R, Benoist C, Mathis D (1992). Intron-exon organization of the NF-Y genes. Tissue-specific splicing modifies an activation domain. J Biol Chem.

[pgen-0030145-b069] Hu Q, Maity SN (2000). Stable expression of a dominant negative mutant of CCAAT binding factor/NF-Y in mouse fibroblast cells resulting in retardation of cell growth and inhibition of transcription of various cellular genes. J Biol Chem.

[pgen-0030145-b070] Bhattacharya A, Deng JM, Zhang Z, Behringer R, de Crombrugghe B (2003). The B subunit of the CCAAT box binding transcription factor complex (CBF/NF-Y) is essential for early mouse development and cell proliferation. Cancer Res.

[pgen-0030145-b071] Farina A, Manni I, Fontemaggi G, Tiainen M, Cenciarelli C (1999). Down-regulation of cyclin B1 gene transcription in terminally differentiated skeletal muscle cells is associated with loss of functional CCAAT-binding NF-Y complex. Oncogene.

[pgen-0030145-b072] Matuoka K, Chen KY (2000). Possible role of subunit A of nuclear factor Y (NF-YA) in normal human diploid fibroblasts during senescence. Biogerontology.

[pgen-0030145-b073] Ying QL, Nichols J, Evans EP, Smith AG (2002). Changing potency by spontaneous fusion. Nature.

[pgen-0030145-b074] Weinmann AS, Farnham PJ (2002). Identification of unknown target genes of human transcription factors using chromatin immunoprecipitation. Methods.

[pgen-0030145-b075] Loh YH, Wu Q, Chew JL, Vega VB, Zhang W (2006). The Oct4 and Nanog transcription network regulates pluripotency in mouse embryonic stem cells. Nat Genet.

[pgen-0030145-b076] Burdon T, Smith A, Savatier P (2002). Signalling, cell cycle and pluripotency in embryonic stem cells. Trends Cell Biol.

[pgen-0030145-b077] Coustry F, Maity SN, Sinha S, de Crombrugghe B (1996). The transcriptional activity of the CCAAT-binding factor CBF is mediated by two distinct activation domains, one in the CBF-B subunit and the other in the CBF-C subunit. J Biol Chem.

[pgen-0030145-b078] Serra E, Zemzoumi K, di Silvio A, Mantovani R, Lardans V (1998). Conservation and divergence of NF-Y transcriptional activation function. Nucleic Acids Res.

[pgen-0030145-b079] Coustry F, Sinha S, Maity SN, Crombrugghe B (1998). The two activation domains of the CCAAT-binding factor CBF interact with the dTAFII110 component of the Drosophila TFIID complex. Biochem J.

[pgen-0030145-b080] Zhu J, Zhang Y, Joe GJ, Pompetti R, Emerson SG (2005). NF-Ya activates multiple hematopoietic stem cell (HSC) regulatory genes and promotes HSC self-renewal. Proc Natl Acad Sci U S A.

[pgen-0030145-b081] Li C, Wong WH (2001). Model-based analysis of oligonucleotide arrays: expression index computation and outlier detection. Proc Natl Acad Sci U S A.

[pgen-0030145-b082] Curwen V, Eyras E, Andrews TD, Clarke L, Mongin E (2004). The Ensembl automatic gene annotation system. Genome Res.

[pgen-0030145-b083] Birney E, Andrews D, Bevan P, Caccamo M, Cameron G (2004). Ensembl 2004. Nucleic Acids Res.

[pgen-0030145-b084] Birney E, Andrews TD, Bevan P, Caccamo M, Chen Y (2004). An overview of Ensembl. Genome Res.

[pgen-0030145-b085] Karolchik D, Baertsch R, Diekhans M, Furey TS, Hinrichs A (2003). The UCSC Genome Browser Database. Nucleic Acids Res.

[pgen-0030145-b086] Chiaromonte F, Yap VB, Miller W (2002). Scoring pairwise genomic sequence alignments. Pac Symp Biocomput.

[pgen-0030145-b087] Schwartz S, Kent WJ, Smit A, Zhang Z, Baertsch R (2003). Human-mouse alignments with BLASTZ. Genome Res.

[pgen-0030145-b088] Bussemaker HJ, Li H, Siggia ED (2000). Regulatory element detection using a probabilistic segmentation model. Proc Int Conf Intell Syst Mol Biol.

[pgen-0030145-b089] Ben-Dor A, Shamir R, Yakhini Z (1999). Clustering gene expression patterns. J Comput Biol.

[pgen-0030145-b090] Yeom YI, Fuhrmann G, Ovitt CE, Brehm A, Ohbo K (1996). Germline regulatory element of Oct-4 specific for the totipotent cycle of embryonal cells. Development.

[pgen-0030145-b091] Smith SB, Ee HC, Conners JR, German MS (1999). Paired-homeodomain transcription factor PAX4 acts as a transcriptional repressor in early pancreatic development. Mol Cell Biol.

[pgen-0030145-b092] Reynolds A, Leake D, Boese Q, Scaringe S, Marshall WS (2004). Rational siRNA design for RNA interference. Nat Biotechnol.

[pgen-0030145-b093] Ventura A, Meissner A, Dillon CP, McManus M, Sharp PA (2004). Cre-lox-regulated conditional RNA interference from transgenes. Proc Natl Acad Sci U S A.

[pgen-0030145-b094] Pfaffl MW, Horgan GW, Dempfle L (2002). Relative expression software tool (REST) for group-wise comparison and statistical analysis of relative expression results in real-time PCR. Nucleic Acids Res.

