# Supplementary material for: Systematic Identification of cis-Regulatory Sequences Active in Mouse and Human Embryonic Stem Cells
Source: PLoS Genet. 2007 Aug 31;3(8):e145. doi: 10.1371/journal.pgen.0030145 (PMC1959362; doi:10.1371/journal.pgen.0030145)
Supplement: Supplementary file 1 [file pgen.0030145.sd016.pdf]

```
Motif 1
ENSMUSG00000042354
>NM_206826 CGTGACG
ACAGTTCCCAAAATCCCGGATTCTTTGTGGTGT------TGAGAAGCTGAAATATCT
ACAGTTTACAAAAACTC-AACTCGTCGTGACGGTACAAATGAGAAGCTTAAGTACCT
ACAGTTTAAAAACACTC-GATTCGTCGTGACGTTACAAATGAGAAGCTTAAGTACCT
ACAATTT-TAAAACCTC-GATTTTCCGTGCTGCTGCAGAGAAGACACTTCAGTATCT
*** **   ***  * *  * *    ***  *         ***  **  * ** **
ENSMUSG00000042354
>NM_206826 CGTGACG
GCACTTTACGGCGGCAGCGTAAGTGCGTGACGCTCGTCAGTGG----CTTCAGTTCA
ACACTTTCCGGCAGGTCCTGGAGCGCGTGACGTTGAACCGCAG----TTCCAGTTCG
ACACTTTCCGGCAGCTTCTTGAGCGCGTGACGTTGGCCCGCAG----TTCCAGTTCG
gcgcTTTACGGCGGTAGCGTCGGTGCGTGACGCTCGGGCTTGGCTGCCTGCGAGTCA
 * **** **** *   *    * ******** *        *     * *   **
ENSMUSG00000010760
>NM_003311 GTCACGA
CTTCCCGGGGCTCCCCGGC--GTGAGTCATCGCGGGCAGGGCTGGCTGC-AGCGCAT
CTTCCCAGGTCTTCCCGGCCCGTGAGTCACGAGGGGCAGGGACCGCTGA-GGTGCAT
CTTCCCGGGGCTTCCCAGCTCCTGAGTCACAGCGGGCAGGGGCTGCTGA-GGTGCAT
CTTCCCGGGGCTCCCTGG---GTGAGTCAGCGCGGGCAGGGCCGGCCGCCTGCGCTT
****** ** ** **  *    *******    ********   ** *   * ** *
ENSMUSG00000030619
>NM_003797 GTGACCG
-GATCTTCGATTTGC-GGAGTTTGGGAGACTTTATTAAAG--GGGGTTTTGTGTCCG
AGATCTCTGGCTTGT----GTTTACGTGACCGC---------GCGGTTATGTATGC-
AGATCTCTGGCTTGA----GTTTATGTGACCGT---------GTGGTTATGTGTGC-
ACATCTTCAACTTCACGGGGTTTGGGTGACTCCTCTGAAGATGGGATTTTAGGTCTG
  ****     **      ****  * ***            * * ** *   *
ENSMUSG00000038335
>NM_018128 GTGACCG
----TCCCGCTGAAGCTC-----CCCTGACTTCTGCCTGCTCCCTCTCCC-----GA
----TCTGGGCAAACCTCAACCACCGTGACCGCTCCCGACTTCTGGCCCCTGGGAGA
TACTTCTGGCCTAACCTAAACCACAGTAACCGCTCCCTACTCCTGGCCCCTCG--GA
----ACCAGCCAGACGCT-----CCTTGATTCCTGCCCGCTCGTTTTCCC-----GA
     *  *    *         *  * *   ** **  **      ***     **
ENSMUSG00000054079
>NM_016001 GTGACCG
CCCAAAC--TGAGG-----------GTGGTCTTTTGATCACCTGCCTCAGTTCCTTG
CTGGAACCACAGGG-----------GTGACCGTTTGATCACCTGTCGCGACTCCGCG
CTGGAACCACACGG-----------GTGGCTGTTTGATCACCTGTCGCGACTCCGTG
CCAAACC--CAGGGTTTCTTTCCTCGTGGCCGTTCGATCCCCTGCCCTG-----TTG
*   * *     **           ***    ** **** **** *          *
ENSMUSG00000054079
>NM_016001 GTCACGTG
AGTTACGTAGAGGGAGGGAGTGCTGGTCACGTGGCAAGAGGCTGCGCAGGCTCCTTCC
AGTGACGTAGC--GCGCGAGCGCCGGTCACGTGGCTTGCGGCGGCGCAGACTCCTTCC
AGTGACGTAGC--GCGTGAGTGTCGGTCACGTGGTTCGAGGCAGCGCAGGCTCCTTCC
CGTTACGTAGG--GAGCGAGTGCTGGTCACGTGCGAGCGGGCCGCGCAGGCTCCTTCC
 ** ******   * * *** *  *********      *** ****** ********
```

```
Motif 2
ENSMUSG00000030619
>NM_003797 GCTCCGC
TAGTCAGGAAGCGCTGTCCT-CCAAGTTCAAGATTAAGGAAACGTGGCATGCACAG-
CACTAACTCGAAGTTGTTCTCCCGAGCTCCGCATCAGGGA----CCGTAGACACAGA
CACTATCTCTCGGTTGTTCTTCCAAACCCCGCATCAGGGACTCTAGACACGGACAC-
TAAAGCGAGAGTATTGTCCT-CCGAGCTCAAGATTAAGGACACTTGGCGTACGCCG-
 *            *** ** ** *   *   ** * ***             *

ENSMUSG00000030619
>NM_003797 CTCAGCGC
GACG-AACCCAGCGCAAGAGTACGCCACGGCGCCTGCGCATCCCCTGACGGGTACTTT
TCCGTAACCCAGCGCGAGAATACGCCTCAGCGCATGCGCACCTGCAGGTGGGACCCGT
TCCGTAACCCAGCGCGAGAATACGCCTCAGCGCATGCGCACCTCCAGATTGGAACCTT
GAC--AACCCAGCGCGAGAATACGCCCCGGCGCATGCGCAGGTCTGAGTCGGTAACTT
  *  ********** *** ****** * **** ******          **     *

ENSMUSG00000038335
>NM_018128 GCTCCGC
cgccgcagccactgcccGGTGCTCGGCTCCGCAGCCATCTTCCGAGCCCCGTATAT
------AGCCACTGCCCGGTGCTCAGCTCCGCAGCCATCTTCCGAGCCCCGTACAT
------AGCCACTGCCCGGTGCTCAGCTCCGCAGCCATCTTCCGAGCCCCG-----
-------------------------------------------GAGCGCCG-----
                                           **** ***

ENSMUSG00000038335
>NM_018128 CTCAGCGC
CCCTCGCCGCCTCCGCGCCTCTCGGCTCAGCGCCCCACGCGCAAGGCGGAGCCCCCGC
CCCTCACCGCCTCCGCGCCTCCCGACTCAGCGCCCCACACGCGAGGCGGAGTCCCCGC
CCCTCACCGCCTCCGCACCTCCCGACTCAGCGCCCCACACGCGAGGCGGAGCCCCCGC
CCCTCGCCGCCTCCGCGCCTCTCTGCTCAGCGCCC-ACGCGCAAGGGGGAGCCCC---
***** ********** **** *  ********** ** *** *** **** ***
ENSMUSG00000038335
>NM_018128 CGCTGCGCT
CGGCTTGGCGAACCCGCTAACACGGCGCT---CTCACGAGCTGCTGGAAAAGACCTTCG
CGTCTCTGCGGACACT----CAGGGCGCTGCGCTTGCAGGCTGGCGGCAAAAAACCTCG
CGTGTCCGCAGAGACT----CAGTACCCCGCGCTCGCAGGCTGGCGAAAAAAACC-TCG
CTGCGTTGCGGAGACGCGGACGTCCAGCT---CTCTAAGGCTGGTCGGGAGACCTATCG
*      **  *  *     *      *    **      ****      *      ***
ENSMUSG00000038335
>NM_018128 CGCTGCGCT
CCACGCGCAAGGCGGAGCCCCCGCCCGCCGCGCTCCCGGCccgccgccgccgccgccgc
CCACACGCGAGGCGGAGTCCCCGCCCGCTGCGCTCCCCGCCTGCAGCCGCCGC------
CCACACGCGAGGCGGAGCCCCCGCCCGCTGCACTCCCCGCCTGCAGCCGCCGC------
C-ACGCGCAAGGGGGAGCCCC--------------------------------------
* ** *** *** **** ***

ENSMUSG00000015176
>NM_004741 GCTCCGC
CGAGGGCGTGATCCCTGACGTCAGAGCTCCGCCTCCACAAGCTGG-CGGCCGGTGGG
CAGGAGCGTGGCATCTGACGTAGAAGCTCCGCCTCC-TTAGTGGG-CGGCTCGTGGG
CGGGAGCGTGACCTCTGACGT-AAAGCTCCGCCTCCTTGGGGGGGAAGGCTAGTGGG
CCAAGGCGTGGCCCGTGACGTCAGTGCTCCGCCTCA-CAAGCTGA-CGGACGGAGGG
*    *****     ******    **********     *  *   **    * ***
ENSMUSG00000015176
>NM_004741 GCTCCGC
CCTCCACAAGCTGG-CGGCCGGTGGGCTCCGCCCTTAACCAAGATGGCGATACGCGT
CCTCC-TTAGTGGG-CGGCTCGTGGGCTCCGCCCCCTG-CAGGATGATGACGCGTGT
CCTCCTTGGGGGGGAAGGCTAGTGGGCTCCGCCCTTTA-CAGGATGACGACGCGTGT
CCTCA-CAAGCTGA-CGGACGGAGGGCTCCGCCCTTAC-CAAGATGGCGACACTTGT
****     *  *   **   * ***********     ** ****  **  *  **
ENSMUSG00000015176
>NM_004741 CCGCTGCGC
GCCCTGTGGAG---CCC-ACCCTTTCCGTTATGCGCC-CGCGCG-GCGCAATGACGTAA
ACCTCGTGTGC---GCCACTCTTTTCCGCTGCGCGCCGCGCGCG-ACTTGATGACG--A
GCCTCGTGGGG---GCCATTC--TTCCGCTGCGCGC--CGCGCG-ACGTGATGACG--A
GCCTTGTGGAGTGTGCCCGCCCCTTCCGCTGTGCGCC-CGCGCA-GTGCGATGACGTCA
 **  ***       **   *  ***** *  ****  ***** *     ******   *

ENSMUSG00000042354
>NM_206826 GCTCCGC
gggCC---CGCCCTGGGCCC--Ggctccgccgcctgcagccccggccgcggtcac
GGGCC---CGCCCCGGGCCC--GGCTCCGCCGCCTGCAGCCCCGGCTGCGGTCCC
```

```
GGGAC---CGCCCCGGGCCC--GGCTCCGCCGCCTGCAGCCCCGGCCGCGGTCCC
gggcc---cgccccgggccc--ggctccgccgcctgcagccccggccgcggtcgc
*** ********* ******************************** ****** *
ENSMUSG00000042354
>NM_206826 CGCCGAGC
tgcagccccggccgcggtcaccgaccgccgcgccccgccccgtggccgccacggctgc
TGCAGCCCCGGCTGCGGTCCCCGACCGCCGAGCCCCGCCCCGTGGCCGCCACG---GC
TGCAGCCCCGGCCGCGGTCCCCGACCGCCGAGCCCCACCCCGTGGCCGCCACG---GC
tgcagccccggccgcggtcgccgaccgccgcgccccgccccgtggccgccacggctgc
************ ****** ********** ***** ****************    **

ENSMUSG00000062223
>NM_003413 GCTCCGC
CGCCGCCTCACCTGC----CAGGCACCCGGCGGCTGCCCAATCAGCGGGGGTCG
GGCTGCCTCACCTGC----CGGGCGCTCCGCAGCTACCCAATCAGCTGGGGTCG
CGCCGCCTCACCTGC----CAGGCGCTCCGCCGCTACCCAATCAGCTGTGGTCG
CGCCGCCTCACCTGC----CAGGCGCCGGGCCACGGCCCAATCAGCGGCGGTCG
 ** **************** *** *    **   *   ********** * *****
ENSMUSG00000034826
>NM_017426 GCTCCGC
CTACGCCCTTGGCGCCTTCCGGCCT-CTTAGGCTAGGTCAGAAAGGAGGCGGAACT-
CTCCATCCTTGCTTGGTTCCTGCCTGCTCCGCTTAGACCGGGAAGGAGGCGGAACTA
CTCCATCATCGCTTGGTTCCTCCCTCCTCCGCTTAGGCCAGGAAGGAGGCGGAACTA
CTGCGCTTCCGGCG--TTCGGGCTT-CCGAGGCGCGGCCAGAAAAGGGCCGGAACT-
** *       *      ***    * * *    *     *   * * ** * * *******
ENSMUSG00000018239
>NM_017665 CGCAGAGG
-AGCCTGTCTGGCCCCGCCTACGTCTAC---GGCCTCCCGG-TTGGAAGCCCCGCCC-
-AGCCCGTCTGGCCCCGCCCAGGACCGCAGAGGTTTCT-------AAGGACCCGCCC-
-AGCTCCTCTGGCCCCGACCAGAACCTCAGAGGTTGTT-------AAGGGCCCGCCC-
-GGCCTCACTGGCTCCGCCCACGCAGGC---GGACGCCGGGGTCGAAGTCCCCGCCCC
* **     ***** *** * *       *    **               *    *******
ENSMUSG00000022474
>NM_002676 CTCAGCGC
ACCTGGCCCTCACGTCACTG-ACCCCGCAGCG-TCCTTGC------------CTGTGT
CCCAGGCCCTCTCGGCCTTCCAGGCCTCAGCGCTCCCCGCGCGGGTTGGCATCCACGT
CCCAGGCCTT--CGGCCTTCCAGGCCCCAGGGCTCCCCGCGCGGGTTGGCATCCACGT
ACCCGGTCCCCACGTCGCAG--CCCCCGAGCA-CCTGCGC------------CCACCT
 ** ** *     ** *          **   **     *    **              *     *
ENSMUSG00000061024
>NM_015169 CTCAGCGC
---ACTAG---CAGTGCGGCCTGCTC---GGGCCAGCTCCG--CCTCTATCTGGCGCC
------AG---CTGGTTAGCCAACCCTCAGCGCCAGTCGGG--CCAACATCCGGTGAC
CTTGTTAGTCTTCATTCAGCCAACCTTCAGCGCCAGTCTAG--CCAACATCCGGTTAC
---AAATG---TAGTCCAGACTCCT----GGGCCAGCACCG--CCTACCTCCGGGCTC
       *          * *  *      * *****     *****     ** **    *
ENSMUSG00000002797
>NM_024051 CTCAGCGC
GGTCGGTGGGGACCGGCAGCCAATCAGGAGAGCGCTCGCTCCTGACTCGACCGGCCCA
CGCCGGCAAG--CCGGCAGCCAATCCTCAGCGCGCTCTCT--------GTGCGGCTC-
CGCCAGCAAG--CCAGCGGCCAATCCACAGCGCTCTCTCT--------CTGCAGCTC-
GTATAGCTGGGACCGACAGCCAATCAGAAGAGCGGTCGCATCCGGCTCGCCCGGCCCA
     *   *  **  * *******    ** **   ** *              * ** *
ENSMUSG00000022247
>NM_018321 CTGAGCGC
GGGGAGTCGGTTTTGTGCAGCCC------GCGCCACCAGCCCC-GCCTCCAACAAA-A
GGGTAACCGGGCTTGCTTAGCACCTCTGAGCGCCACCAACCTG-GCTTGCAGGAGA-A
GAGG-------CCTGCTTAGCCCCTCTGAGCGCCACCAACCTTAGCCCGCCACAGACA
GCCAAACCGGGCCTCCCCGCCCC------------CCAACTCT-GCCGCTAACGGA-A
*            *      * *             *** *     **          * *


Motif 3

ENSMUSG00000030619
>NM_003797 GTACCGT
AAGCGT----CTAGAGTATAATTCGGTAGCGCTTTGAAATCCACCCTG-GGATTCGG
AGGCGTGTCCGTAGGCACTGATTTGGTACCGTTTTGAAATCCTCCCCGAAGCTTCAG
AGGCGTGTCCGTAGGCACTGATTTGGTACGGTTTTGAAATTCTCCCCAAGGTTTCAG
ACGCAT--CCCGACAGTCCGCCTCGCTGGTGCTTTGAAATCCACCCCG-GGCTACGG
* ** *       *          * * *    * ******** * ***     * * * *
```

```
ENSMUSG00000030619
>NM_003797 TACGGTAT
TAAGGGT-TGTCACTCTAAGACCCTTGTGGAATTTTCAGGATGGAAAGT-------GA
GGGGGCTGTGTCACTGT-AGAGCACTACGGTATCTTCAGGATGGAAAGT----ATAGA
AGGGGTTGTGTCACTGT-AAAGCACTACGGAATCTTAAGGATGGAAAGT--ATATAGA
TTGGATCGTGTCTCTCCTCGACCCTTACCGATTGTCCAGCACGGGAAGCGCAGTTACA
   *    **** **     * *  *   *  * *  ** * ** ***         *
ENSMUSG00000022940
>NM_153681 GTACCGT
aaaagggtagtgataatTGAAGGGATCTCGT--GTTTGCTGAGTGTTCCGTGTGCC
-----------------AAAAGGGGTACCGTTTGTTTGCTCAGT-TTATGTATGC-
-----------------TGAAGGG-TACCGTTTGTTTGCTCAGT-TGATATATCC-
aaaaggatggtgataaATGAAGGGAATTTGC--ACTTGGT--GTTTGATATGTCC-
                   *****     *     *** *  ** *    * * *
ENSMUSG00000022940
>NM_153681 GTACCGT
GGCGTCCAGGGCGCCACTGGAAAAGGCACGGCGCACTGGTACCAGCCCAAATTCACC
GGAAACTACAA-GCTTCTGAGAAAGGTACCGTGCAGTGGTACCGACACAC-------
GGAAACTACAA-GCTTCTGGAAAAGGCACCGTGCAGGGGTACCAGCACAC-------
GGCGTCTACGGCGCTTCAGGAAAAAGCACTCTGCACCGTCACCAGCCAAAATTCACC
**   * *    **  * *  *** * **   ***  *  ***  *  *
ENSMUSG00000022940
>NM_153681 TGGTACCG
CGCCACTGGAAAAGGCACGGCGCACTGGTACCAGCCCAAATTCACCTACGTCTCGGCT
-GCTTCTGAGAAAGGTACCGTGCAGTGGTACCGACACAC-----------CCTCGGCT
-GCTTCTGGAAAAGGCACCGTGCAGGGGTACCAGCACAC-----------CCTCGGCT
CGCTTCAGGAAAAAGCACTCTGCACCGTCACCAGCCAAAATTCACCTACGCTTTGGCT
 **  * *  *** * **   ***  *  ***  *  *              * ****
ENSMUSG00000022247
>NM_018321 GTACCGT
aa-AAAGGCGCCT----ACTCTGCGCATTCGGCCCCGAG-----GGATGCTCCT
AA-AAAAATGTCTTCACTCCCGGTACCGTCGGCTCCGAGAAGAAGGATGCTCCT
AA-AAAAATGTCTCCACTCCCTGTACGTTCAGCTCAGAG-AGAAGGATGCTCCT
AA-AGAGACGCCT----ATTCGGCTCCTGCGGCCCCAGG-----GGATGCTCCC
**** *   * **       * *  *   * ** *   *     *********

ENSMUSG00000022247
>NM_018321 TACGGTAT
tggaggaaaa-----ttatctaagctacggtttcctcgtct-------cc-gtaagag
----------------TCTCAAAACTACGGTATTCT----------------------
----------------TCTCAAAACTACGGTTTCCTTGTCTGATACAGCC-GGAAAAT
TGGGGGGGGAACGACTCCTCCAAGCTACGATTTCCCCGCCT-------CTAGCAAGGG
                  ** ** ***** * * *
ENSMUSG00000038335
>NM_018128 ACCGTACC
CGAGCCCCCCGGGGTTCTTGACTCGACTTTACCTCCTTCTTCACGTTCCACAGCAGTT
CGAGCCCCTTGAGACCTTGCCCCGGACCGTACCTCCTTCTTCACGTTCCACAGCAGCT
CGAGCCCCT--GAGACCTTGCCCCGACCGTACCTCCTTTTTCACGTTCCACAGCAGCT
CGAGCCCCCGGGGGTCCTTCCCCGGACTTTACCTCCTTTTTCACGTTCCACAACAGTT
********         *   *  ***  ********* ************* *** *
ENSMUSG00000054079
>NM_016001 ACCGTACC
CCTCTGCAGGCTCCGTACTCCAAGGACCGTACCAAGACGAAGGGTGGAAAGCGTA--T
CCTCGGCAGGCGCCGTACTCTAAGGACCGTACCAAGACCACAAGTCGATGAGGTAATT
CCTCTGCAGGCTCCGTACTCTAAGGACGGTACCAAGACCAGGAGTCGATGAGGTAA-T
CCTCCGCAGGCTCCGTACTCCAAGGGCCGTACCAAGACGAGGGGTGGGAGAGGCAA-C
**** ****** ******** **** * ********** *   ** *     * *
```

```
ENSMUSG00000022474
>NM_002676 TGGTACCG
ACC-------GAGTGTGGGATGCGGCGGTACCCGGCCCCCTGACATCCGG-GGCCACC
ACCCTGTCGGAAACGTGGAGTGCAGTGGTACCGG--CTCCTG---------------T
ACCCGGTCGGAAACGTAGAATGCAGTGGTACCGG--CTCCTG---------------T
ACC-------GAGTGTGGGACGCTGCGGTCCGCGG-CCCCTGACGCCCCACGGCCGTC
***        *  ** *   ** * *** *  *  * ****
```

Motif 4

```
ENSMUSG00000015176
>NM_004741 CGCGGGAA
AAAGGTACCTCCCCCAGAAAGACTGCGCGGGAAGTCCCGAGTTTTCCCGCCAC-GCCG
AGAAGCCCATTCAGCAAAGGTAATGCGCGGGAATTTGCAAGTTTTCCCGCCTCGGACA
AGAAGTCCATTCAGCAGAGATAATGCGCGGGAAGTTGCAAGTTTTCCCGCCTCGGACG
AAAGGTGGCTCTAGCAGAAAGG--GCGCGGGAAGCCTGAAGTTTTCCCGCCACTGGCC
* * *    *    ** *      *********      ************ * * *
ENSMUSG00000062223
>NM_003413 CGGGAATT
GGAGCGCGAAACTGCTGCGCTCTCCCCAGGATCTGCGCCCTGCAGCTACAGAGGGGAC
GGAACACAGATTTGCCCCACTCATGCGGGAATTTGTTCTCGGTACCTACGGAAGGAAA
GGAACACAGATTTGCCCCACTCACGCAGAAATTTGTTCCCGGCACCTACGCAAGGAAA
TGAACCTGGAGTTGCTATGCTCGCCCCACGATCGGCGCCCCGCGGCCGCCGAGAGGGC
 ** *    *  ***    ***   *    **  *  * * *   *  *  *  *
ENSMUSG00000054079
>NM_016001 CGGACGGA
GAACTCCCACACGGGGCGGGGCTGGGTAGGGGAGCGGGAGTTACGTAGAGGGAGGGAG
GAACTCCCACATGGGGCGGGGCCGCCGGACGGAGTGGGAGTGACGTAGC--GCGCGAG
GAACTCCCACACGGGGAGGGGCTGCCGAACTGAGTGGGAGTGACGTAGC--GCGTGAG
GAACTCCCCTACGGGGAGGGGCTGGGGAGGGGAGTGGGCGTTACGTAGG--GAGCGAG
********  * **** ***** *       *** *** ** ******   * * ***
ENSMUSG00000042354
>NM_206826 ATTCCGTT
------------------AAAAAATAC-------------------GGAAAGAAAATC
------------------AAAAAATATTCCGTTTGGGCGATCATTGGGAAAGAAAAAA
------------------AAAGA-----------------------GGAAAAACAAAA
CTGAAACACAGTCATCTAGAAGAAGGt-------------------aaaaaaaaaaaa
                   ** *                         *** * **
```

Motif 5

```
ENSMUSG00000020464
>NM_033109 CGTCCGAC
----GGCACCGCGGAAACGAAACTCCATCAGGCTCCGCCCCACGGTCTGCGGAGTGAG
----GGCTCCACGGAAACGAAACTGCGTCCGACTCCGCCCACAGGAGTGCTG-----G
----GGTTCCACGGAAACGAAACCGCGTCCGGCTCCGCCCACCTGCGGGCTG-----G
CCCCAGCACCGCGGAAACGAAACTGCCGCAGGCCCCGCCCCGC-GCCTGCGGAGCGAG
     *  ** ************  *  * * * ******    *   ** *     *
ENSMUSG00000061024
>NM_015169 GACGTCCG
CTTTCTTTTCCGGATTGGGCATCCCGGCATCTGCACGTGGTTATGCTGCCGGAGTTTG
TTTCCTCTTCCGGATTACGGGTCCGGACGTCCGCACGTGGT-----TGCCG--GTTTA
TTTTCTCTTCCGGATTACGAGTCCGGACGTCCGCACGTGGT-----GGCTGGTTTTTG
CTTTCTCTTCCGGAACGAGCGTCTCGGCGGCCGCACGTGCTTGCGTTGCCGGGGCGAG
 ** ** *******    *  **  * *  * ******* *      ** *
ENSMUSG00000022247
>NM_018321 GTGACGTC
TCTCGGGGTCCCCGAGTGCGGAAGGTGGGGTCTGGACGCCCGAGAGCCCTTCTCAGC
----GGGAGCCCCTGGCG-----GGTGACGTCC-GACCCCTCAAAGCCCCTGCCACC
G---GGGAGCCCCGGGCGCCAGGGGTGACGTCC-GATCCCGTAAAGCCCTTGTCAGC
TCCCGGGATCCCGGGACGCGGAAGGCGGGATCCGGGCGCCAGGGAGCCCTCCGCAgc
    ***  ***     *     ** *   **  *   **    *****    ** *
ENSMUSG00000020089
>NM_021129 GTGACGTC
AAGCTGTTTCCCTCAAATCAAACAGATGACGTCAGTCTGGAAA-CAAGACTGAGAAAG
AAGCTTTGCCTCCCTAGACAGACAGGTGACGTCTAGCTAAGGA-CCTGGTGGGAGAAC
AGGCTTTGCCTCCC----CAGACAGGTGATGTCCAGCTGAGGA-CCTGGAGGGAGAAC
AAGCTGTTCCCCTCGAGTCAAACAGGTGACGTCAGTCTGGGAAGCACGAGTGAGAAAG
* *** *  * * *    ** **** *** ***   **    * *  *   *   **
```

Motif 6

```
ENSMUSG00000035443
>NM_199298 CAATCA
CTACAGTTCCTTTTTCTTGTTTATTCAGGCAAGCCCC-----------TAGGCTG
TTTCAGTCCCTTCCACTTATTTGTCCAATCAAGAACC-----------GACGCTG
TTCAAGTCTCTTCCACTTGTTTGTCCATTCAAGCACC-----------GACGCTG
CCAGAGCCTCTTCTGTGTGTTCACTGCAGCAGGCTCCTTGGGGCTGCTGGGGCTG
    **   ***     * **        ** **  **              ****
ENSMUSG00000035443
>NM_199298 AGTGATTG
TTATAAACATT--CTTGGCAGAGAAG-----TCACAAAC---------TGGCCG----
TTCTGGTTTTTAGTTTGGTATTTAAAGTGATTGACAAATCTAAATATTTGGCTT----
TTCTGGATTTTTGTTTGGTATTTTAAGTGATTGACAAATCTCAAGAACTGGCTT----
----AAACCGAGGCTGGGCA-----------TCCCTCGC---------CGGCCTCGTA
              * ** *           *  *              ***

ENSMUSG00000035443
>NM_199298 CAATCA
CAAATTACTGTTCAGAGCCTAAAGACACTCAAGTTTGTCAGCTCTGG-TAGAGTTT
CAAGTTTTCTGCCAGGTTTTTAA-ACAATCA-GTTTGTAATCTCAGA-TAGGGTTT
CAAGTTTTCTGCCAGGCTCCTTAAACAACCA-GTTTGTAATCTCAGA-TAGGGTTT
CACAGCATGGCCTGGTGCCTGAGGACGCTCG-GTCTGTTAACTCTGGGTAGTTGCC
**            *         **   *  ** *** * *** *  ***
ENSMUSG00000018239
>NM_017665 CAATCA
caataaatattaatttatttagttcctaccgttctgtgccagacattgggtataaa
AAATAA--------TTATTTTAGTACAATCACTC----ACAGACAGTTTCTCTGAA
CAGTAA--------TTACTTTAGTACTAGCATTCCTTGACAGT--TTCAGTGTGAA
caataaataac---tgattcatcatctgtttctcgctgtcagattttgggtgtgaa
 * ***        * * *      *      **     ***    *   * * **
ENSMUSG00000030619
>NM_003797 CAATCA
TTGGTAGCAACTGG--AAGGATAATCATTCATTACAATAAATAGTCAACAGAATAA
GTGGCACCAACCGGTTGAATATAACCAATCATTGTAATAAGTATTTATTAGATAAA
GTGGCACCAACCGGTTGAATATAACCACTCGTTGTACTAAGTATTTATTAGATAAA
TCGGCA-CAAGTGGGTGAGAATCATCACTGATTAAAATAAATATTCATCGGCTTAG
  ** * ***  **   *  ** * ** *  **  * *** ** * *   *   *
ENSMUSG00000022940
>NM_153681 CAATCA
CCAAAATGATTTGAGAATTTTGAGGCACACAAAAGGGAGAAAATTGAGTGCCCGTG
TCAAA-TAGTTTGGAAGATATGAGGCAATCAAGTGGTAGAAAATTGAATGCCCACA
TCAAA-AAGTTTGGAAGATATGAGGCAATCAAGTGGTAGAAAACTGAATGCCCACA
TCGAAAGGATGTAGGAATTATAAGGAAAACAGGAGA---AAGATTGAGTGTCCCC-
 * **    * *   *  * * *** *  **    *    ** * *** ** **
```

```
ENSMUSG00000062223
>NM_003413 CAATCA
TGCCATGTTCTCCGCTCCGCGCTGCCAATCAGCCTCGCGGCGGCCAATGGGCAGTC
TGCCATGTTCTCCGCTTCGCGCTGCCAATCATTGTGTCGGTGGCCAATGGGCGACA
TGCCATGTTCTCCGCTTCGCGCTGCCAATCAGTGTGGCGGCGGCCAATGGGCGGCA
TGCCATGTTCCCCGCTCCGCGCTGCCAATCAGCGTGGCGGCGGCCAATGGGCTGCA
********** ***** **************    *  *** ***********

ENSMUSG00000062223
>NM_003413 AATCAGC
---CAGGCACCCGGCGGCTGCCCAATCAGCGGGGGTCGCTCGGCCGCGGCTGCCA
---CGGGCGCTCCGCAGCTACCCAATCAGCTGGGGTCGCCCGGCAGCGGCTGCCA
---CAGGCGCTCCGCCGCTACCCAATCAGCTGTGGTCGC-CGGCAGCGGCTGCCA
---CAGGCGCCGGGCCACGGCCCAATCAGCGGCGGTCGCTCGGCTGCAGCTGCCA
**** *** *    **  *  ********** * ****** **** ** *******
ENSMUSG00000029922
>NM_013446 CAATCA
TTTCCGGAGGAGACGTCCCCTCCACCAATGAgcgg-cgccgtgggcgggccgcggg
TTTAGGGCGGAGCCTAAGCCATCACCAATCAGCGT-CGCCGGAGGAGGGCCGCGGG
TTTAGGGCGGGGCCTGACTCCTCACCAATCAGCAT-CGCCGAAGGAGGGCCGCGGG
CACGGGGCGGAGCTTCCGCGTCGGCCAATCAGAGGGCGCTGGGGGCGGGCCGCGGG
     ** ** *             ***** **    *** *  ** **********
ENSMUSG00000024406
>NM_002701 AATCAGC
CACCAGACCCAGCTTG--GGGTTGGTTTGAGCCCC-TTTTCCCACCC----------
CACCAG--------TGATGCGTGAAAATCAGCCCC-------CCCCCAAAAAAAAAA
CACCAG--------TGTTGGGTGAAATTAAGCTTTGTTTTTTCCTTC--------AA
TACCAGACCCAGCTTG--GGGT----TTGAGACCC-TCTTCCCACC-----------
 *****        **  * **     * **           *
ENSMUSG00000054079
>NM_016001 AATCAGC
CTTGTGTCAAGTCAAGTGG-TTAC-AGCCAAGAGGAAAAGCAGAAGTTCCATTCAGC
CGCGCGGCACGACGCGGGGCTCAG-AATCAGCAGGGCG---AGAAGTTTCTTTCAGC
CTCGCGGGAAGACGCGGGGCTCAG-AGTCAGCAGGAAG---AGAAGTTTCTTTCAGC
CTCGAGGGAGGTCGAGTGGCTAACAAGTCAGGAAGAGGAGCAGAGGTTTCATTCAGT
*  * *  * * *  * ** * *  *  **  * *      *** *** * *****
ENSMUSG00000054079
>NM_016001 AATCAGC
AGCTGTTGGG-TTTGAAGGCTATTAGACTAGTAGTAGAGCAAGTAGAAA-TATCAT
ACTTGATGGGCTTTGAAAGATCTTAAATCAGCAGT---GCTGGTGGATA-CCT-GT
CTTTGATGGGCTTTGAGAGATCTTAAATCAGTAGCA--------------CTTTGG
GTTTATTGGACTTTGAGGGATCATAAGTTGGTAATAGACCAGCAAAAAATATTAGT
   *  ***  *****  * *  **     * *                    *
ENSMUSG00000002797
>NM_024051 AATCAGC
TGTGAGGAGTGCCTGTCCACCCCTCAATCAGAGGAAGCCTTGAGCAAGGAGGGGCGG
-----------------------CCAATCAGCGGC-GCCTCAGGCAGGG---GGCCG
-----------------------CCAATCAGCGAC-GCCTCAGGCAAGG--GGGCCG
TCTCCGTAGCGCCCGCCCACCCGCCAATCACAGGGAGCCTCGGGTATCT--GGGCGG
                        ******  *   ****   * *      *** *
ENSMUSG00000002797
>NM_024051 TGATTGGC
aattgaaagccccag---aatgtcccgattggctaagttttggccacgtacccatgcc
AATTGAGAGCCCCTGCGAGGTGCCCTGATTGGCTAAATCTCA-CCATGAATCCACGTC
AATTGAGAGCCCCTGTGGGGTGCCCTGATTGGCTAAATTTCA-CCAAGTATTCATGTC
aattcagagtcccaggtgggtgtcctgattggctaa--cttggccatgtgcccacgcc
**** * ** *** *      ** ** **********   *   *** *    ** * *

ENSMUSG00000020089
>NM_021129 TGATTGGC
CTGGGTGTTTTACCAGACCTGATAATGACTGGCTCTCTGAATACATTCGATGAAACAA
CTGGGCGTTTTATTATAGAGGGGACTGATTGGCTCTCTGAATATGTTCAATGGA-TCA
CTGGGCGGTTTACCATAGTGATTACCGATTGGCTCTTTGAACTTATTCAATGGA-TCA
CGTGGCATTTTATTAGAGTTGCTACTGACTGGCCTTCCAAACACACTCAATGAAATAA
*  **   ****  * *      *  ** ****  *   **      ** *** *   *
```

```
Motif 7
ENSMUSG00000010760
>NM_003311 CTACTATT
GGGAAGAGCCGGCTTTCGCTTCCTGT--CTATTAAACCCTTTAAAGGAAGAGGGT---
TGGAGGAGCTG-CGTCTGCTTCCTACTACTATTAAACC-TTTAAAGGCAGAGGGT---
TGGAGGAGCAG-CGTCTGCTTCCTGCTACTATTAAACC-TTTAAAGGCAGAGGGT---
GGGAGGAGCC--CGGCAGCGCTCGGCTCCTATAAACCC-TTTAAAGGAAGAGAGTTTa
 *** ****   *    **   *     **** ** ** ******** **** **
ENSMUSG00000041438
>NM_032830 CTACTATT
CCCCTCTTCTCCACCCCCCTCCAAATTAC-----------------------------AA
CCAACTTCCTTCAAACTATCCCAAACTACTATTG------------------------GA
CCCCATTTCTCCAACTCCCTTCAAACTATCCC--------------------------AA
CCTCTCTTCCCCA-CCCCCTCCGAATCACCCCCAGAATTTCGCTGGGACTTCCTACCA
**    * *  **        * **  *                             *
ENSMUSG00000022247
>NM_018321 TGACAATA
-------CTCTTTAGTAatagcatatcgcagtggaaaaaagagcatatattctggaaa
----------------AAACGTGCATGACAATA---------------ATACTGTAAA
AAACATGCATTTCAATAAT--TATATTGTA--------AAGAGTAAACATTCTCG---
------------aagtagttgcc---------------aaaaccatatactctggaaa
                *                               *  **
ENSMUSG00000038172
>NM_152574 ATTACAAT
agtcctggcacatagtaaggactcaagaactattag----ctgttTTTACAGTTATCA
AGTCCTAGTACACGATTAGTTCTCAATTACAATTAGTTTCCTGTTTTTATGTTTACA-
AGTCCTAGTACATGATTAGTTCTCAGTTAAAACTAGTTTCTTGCTTTTATGTTTACA-
agtcctgatacatagtaagtactcaataaatactag----ctATTTTTATCATTATCA
******   ***   * **  ****   *  * ***     *  *****   ***
ENSMUSG00000012396
>NM_024865 ATTACAAT
GGGTTACTCTGCAGCTA-CTTTTGCATTACAATGGCCTTGGTGAGACTGGTAGACGGG
GGGTCACCTTACAGCTT-CTTTTGCATTACAATGTCCATGGTGGACCCTGCAGGTGGG
GGGTCACTTTACAGCTT-CTTTTGCATTACAATGTCCTTGGTGGGCCTGGCAGGAGGG
GGACTACCCGGCAGCGACCTTTTCCATTACAATGGCCTCGGTGAGACTGGCAGACAGG
**   **    ****    ***** ********** **  ****   *  * **   **
```

```
ENSMUSG00000062223
>NM_003413 GTATTGTT
TTACTGCGT--TGTATATGTTTGCTGTGTCG-TGCCCGGTGTGTAATTCGGGAAG---
----TGTGT--TGTACATGGCTGTTGTATTGTTGTCTGTTCTATCACGGGACAAGGG-
----TGTGTTGTGTACATGGTTGTTGTACTG-TGTCTGTTTTATCG-----------
TTACTGTGT--TGT--ATGTTTGTTGAATTG-TGGCTGGTGTGTCATATATGGGGGA
    ** **  ***  ***  ** **    * ** * * * * *
ENSMUSG00000054079
>NM_016001 GTAACAAT
T---GTTAGTTGCCAGATACCCTTTCTGGCAATGGCAAACCCTGCGTGATTGAT----
TTTCTTTGGTTGCTAG----CTCTTGTAACAATTGCAAATCCTTCCTGATTG------
----TTTGGATGCT---TACTCTTTGTAACTATTGCAAACCCCTCGTGATTG------
T---GCTGGG-GCCACGTCCTTTTCCTGACGGT-GAAAACCCCGCGTGCTTTTTATCG
      * *  **          *  *  *  * * *** **  * ** **
ENSMUSG00000015176
>NM_004741 TATTGTTA
taaag-ggaatttgaaTCGAGC----CCGCTTGTCCACTCATTACTCTGGAATCCGA
TGAAA-GGCATGTGAAGCACCCATTATTGTTAGTCCATTCAGTACTGTGAGGTCTCA
TGAAA-GACATGTAAATCGAGT----TCTCCATTCCTTTGATTATCGTGGGGTCTGA
taaaagggaatttgaa-TCAAC----CTGCCAGTCCACTC--TACTCTGGAATGCGA
* **  *  ** * **                 ***  *   **   **   *   *
ENSMUSG00000024406
>NM_002701 TATTGTTA
-TAAAAACAGTGCTCATTCCAGGATTATTGTTCCTGAAAGTCTAGGGTGTGACTC---
TAAAAAATGGTGCTTATCCTGGAGTTATTGTTACTGAAGAGGTTGGGTGTGACTG---
-GTAAAATGGTGCTTATCCAGGAGTTATTGTTACTGAAACGCTTGGGTGTGACTG---
-TAAAAACCGTGCTTATCCTAGGGTTATTGTTCCTGCAAGTGTAGGGTGTGACTG---
   ****  ***** ** *  *  ******** *** *    * ********** ***

Motif 8
ENSMUSG00000042354
>NM_206826 CACGTGGC
CTCGTCAGTGG----CTTCAGTTCACACGTGGCGCCAGAT-------GTGTTTGTGC
TTGAACCGCAG----TTCCAGTTCGCACGTGGCGCCCGATCCCGAGACCTCCTCTGC
TTGGCCCGCAG----TTCCAGTTCGCACGTGGCGCCCGTACCTGAGACCTCTTCTGC
CTCGGGCTTGGCTGCCTGCGAGTCACACGTGGCCGGGGTT------GCTGCTGCTGC
 *        *     * *   ** ********    *                 ***
ENSMUSG00000054079
>NM_016001 CACGTGGC
TTACGTAGAGGGAGGGAGTGCTGGTCACGTGGCAAGAGGCTGCGCAGGCTCCTTCCGG
TGACGTAGC--GCGCGAGCGCCGGTCACGTGGCTTGCGGCGGCGCAGACTCCTTCCGG
TGACGTAGC--GCGTGAGTGTCGGTCACGTGGTTCGAGGCAGCGCAGGCTCCTTCCGG
TTACGTAGG--GAGCGAGTGCTGGTCACGTGCGAGCGGGCCGCGCAGGCTCCTTCCGA
* ******   * * *** *  *********      *** ****** *********
ENSMUSG00000054079

>NM_016001 CCTGGCAT
TTCCTCCACTTCCCTGCTGTTCTTTCTTGGCACACAAAATATAGGCCAATGAGCAGTG
TCCCTCCACTTCCCTGATGTTCTTTCCTGGCATACAAAATATAGGCCAATGAGCAGTG
TTCCTCCACTTCCCTGATGTTCTTTCCTGGCACACAAAATATAGGCCAATGAGCAGTG
TTCCTCCACTTCCCTGCTGTTCTTTTCTGGCACACAAAATATAGGCCAATGAGCGGTG
* ************** ********  ***** ********************* ***
ENSMUSG00000054079
>NM_016001 ACGTGG
ATTCGATTTCGTTGATGGAATCAATTCGTGGGCACGGGCCTGGGCATCTCCGTTGT
----------GTGGATGGAACCAATACGTGGGCAGAAGGC-GTAAAGCTGCATTTT
----------GTGGATGGAACCAATACGTGGACAGACGGC-GTAAAACTGCATTTT
----------GCTG-TGGAATCAGAATGTGCGT-------------------TTTT
          *  * ***** **    ***                      ** *

ENSMUSG00000015176
>NM_004741 CGTGGCAT
CGAGTTTTCCCGCCAC-GCCGAGGGCGTGATCCCTGACGTCAGAGCTCCGCCTCCACA
CAAGTTTTCCCGCCTCGGACAGGAGCGTGGCATCTGACGTAGAAGCTCCGCCTCC-TT
CAAGTTTTCCCGCCTCGGACGGGAGCGTGACCTCTGACGT-AAAGCTCCGCCTCCTTG
GAAGTTTTCCCGCCACTGGCCAAGGCGTGGCCCGTGACGTCAGTGCTCCGCCTCA-CA
  ************ * * *    *****     ******    **********
```

```
ENSMUSG00000041438
>NM_032830 CACGTGG
AAAACGTGTGTGGATGCTATGCATACACGTGATAC-TTCCCATCACATTTTAGGATT
AAAACGTGTTTAGAGACTATGCATCCACGTGGTACGTTCCCATCACGTGGCAGGATT
AAAACGTGTTTAGATACTATGCATACACGTGGTATGCTCCCATCGCGTGGCAGGATT
AAAACATGTGTGGATACTATGCATACACGTGGTACTTTCCCATCACATTTTAGGATT
***** *** * **  ******** ****** **   ******* * *   ******

ENSMUSG00000041438
>NM_032830 CACGTGGC
ATGCATACACGTGATAC-TTCCCATCACATTTTAGGATTCCCACGTT-GGAGGGAATC
ATGCATCCACGTGGTACGTTCCCATCACGTGGCAGGATTCCTCCACT-GGAGGGAATC
ATGCATACACGTGGTATGCTCCCATCGCGTGGCAGGATTCCTCCATT-GGAGGGAATC
ATGCATACACGTGGTACTTTCCCATCACATTTTAGGATTCCCATGATGGGGGGGAATC
****** ****** **   ******* * *   ********     * ** *******
ENSMUSG00000041438
>NM_032830 CACGTGG
CAGAGC--CTGAGAGCGC--GCGCGCACGTGGGGC----CGGGGCGGAGAGAGG-CG
CGGCGCTGTCGAGTGCGCAAGCGCGCACGTGGGGC----CGGGGCGGAGAGAAG-CG
CGGAGCTATAGAGTGCGCACGCGCGCACGTGGGAC----CGGGGCGGAGAGAGG-CG
CAGAAC--ATGCAAGCAT--GTGCATATCTGTGGA----CATGGCAGAGAGAGG-GG
* *  *    *   **    * **  *  ** *  *****  *** ****** ** *
ENSMUSG00000038335
>NM_018128 CACGTGG
CCGGAAGCAGTGCTGACTCCGTACACGCGCG--CCTGCCCTCCCC----C---ATC
CCGGAAGTAGAGTTCACTTGGTGCACACGTGGAC-----CTGCCCGGGAC---ATG
CCGGAAGTAGTGTTTACTCGGTGCACGTGGG--C-----CTGCCAGGGAC---GC-
CCGGAACTAGTGCTGATTGCGTGCACGTGAG--C-----CTGTCT----CTAGATC
******  ** * * * *  ** ***  * *  *     **  *     *
ENSMUSG00000061024
>NM_015169 CACGTGG
TCCGGATTGGGCATCCCGGCATCTGCACGTGGTTATGCTGCCGGAGTTTGGGCCGCC
TCCGGATTACGGGTCCGGACGTCCGCACGTGGT-----TGCCG--GTTTAGGGTGCT
TCCGGATTACGAGTCCGGACGTCCGCACGTGGT-----GGCTGGTTTTTGGGCT-CT
TCCGGAACGAGCGTCTCGGCGGCCGCACGTGCTTGCGTTGCCGGGGCGAGAGCCGGC
******    *  **  * *  * ******* *      ** *        *
ENSMUSG00000012396
>NM_024865 ACGTGG
--------------------------------------TCAAAGAGTTTTGTCTTC
AGAGAGAGAGAGAGAGAGAGAGACTACGTGGT--TATTTCAAAAACTTGAGTGTGG
-------------------------AAGAAGTTGTATTTCAAACACTTGAGTGCTG
--------------------------------------TC--AGACTTGCATCTCT
                                      **  * * **   *

ENSMUSG00000022474
>NM_002676 ACGTGG
GGACACCCGGG-GACC-------GAGTGTGGGATGCGGCGGTACCCGGCCCCCTGA
AGACACCTGCGCGACCCTGTCGGAAACGTGGAGTGCAGTGGTACCGG--CTCCTG-
AGACACCCGCGCGACCCGGTCGGAAACGTAGAATGCAGTGGTACCGG--CTCCTG-
GGGCCCTCCAG-GACC-------GAGTGTGGGACGCTGCGGTCCGCGG-CCCCTGA
 * * *    * ****        *  ** *   ** * *** *  *  * ****
ENSMUSG00000022247
>NM_018321 ACGTGG
AATAGACGAATGACTTGATAA-TGCATGAGCAACATTAGAGATCATGCTGTGCAC
-------GAATGGTTTAATCATTAAACGTGGAACACTAGGGCTCTTCATCTTCAT
A------GGACAATTTAATAA-TGCATGCATGATAATTGAGTTGATACTAT--AC
AACAGCCAAACAATCTGATAA-TGCATCAGTGATACCGGAGGTTACACTATGCAC
         *     * ** * *  *      * *   * * *     * *  *

ENSMUSG00000035443
>NM_199298 ACGTGG
GCTGCACAAAGGCAGCCCAAGAAAAAACTTTTCTCGAAGAGCCTGTTTAGTACTC
GCTGCA-AAAGGCAGCAGGTAAAGAACGTGGTCTTGTAGGGCGTGCTTTATTTC-
GCTGCA-AAAGGCAGCACGTAAAGAACGT--TTTTGTAGGGCGTGCATTGTTTA-
GCTGCAAAAATGCAGCCGGGTAAAGGT----TTCTCTGGGATCTGTTAAGTGCTC
****** *** *****     **        *      *    **     *
ENSMUSG00000002797
>NM_024051 ACGTGG
agtaaatggcagcgtcgggatttaagcccaggcatgtaacttccaTGTAACAGGGA
AGCAAATGGCA-TGTCCGGATCTGAACGTGGG---------------------AGA
AGCAAATACCA-CGCCGGGATTTGAATGTAGG---------------------GTA
agtaaagggcagcgtcgggatttgaaccccgc-ccgctagttccaTCCA----GGA
** ***   **  * * **** * *     *                        *
```

```
Motif 9
ENSMUSG00000054079
>NM_016001 ACGACAT
AAAAAAGGAGCAACTTAACCAG-----GACATGAATGAGACTGAAGGGTCTAATAAG
AAAAAGGGAGCAACTTAACCAGGACACGACATGAATGAGACTGAAGGGTCTAATAAG
AAAAAGGGAGCAACTTAACCAGGACATGACATGAATGAGACTGAAGGGTCTAATAAG
AAAAAGGGAGCAACTTCACAAG-----GACATGAATGAGACTGAAGGGTCTCATGCG
***** ********** ** **     ************************ **  *
ENSMUSG00000054079
>NM_016001 CACGACG
GGAAAGCGTA--TTGGGGCTTGTGTCAAGTCAAGTGG-TTAC-AGCCAAGAGGAAAA
CGATGAGGTAATTGAGGGCGCGCGGCACGACGCGGGGCTCAG-AATCAGCAGGGCG-
CGATGAGGTAA-TGAGGGCTCGCGGGAAGACGCGGGGCTCAG-AGTCAGCAGGAAG-
GGGAGAGGCAA-CCGCGGCTCGAGGGAGGTCGAGTGGCTAACAAGTCAGGAAGAGGA
 *     * *      ***  * *  * * *  * ** * *  *  **  * *

ENSMUSG00000038335
>NM_018128 CACGACG
CACACTGCGAAGTGGGAAAAGTTCCCAGCGCGGCTTGGCGAACCCGCTAACACGGCG
AGGACTGCAGCGTGGGAAAAGGCACCACGACGTCTCTGCGGACACT----CAGGGCG
ACGACTGCAGCGTGGGAAAAGTTACCACGACGTGTCCGCAGAGACT----CAGTACC
CCAACCGCCGCGTGAGAAAAGTTTTCTCCGCTGCGTTGCGGAGACGCGGACGTCCAG
   ** **   *** ******    *    *      **  *  *     *
ENSMUSG00000024406
>NM_002701 CACGACG
ATCAACTGCTGGCAGCCCTGGCAGGCAGGGGCCAGGTGGGAAGTGGAAGCTGGATTT
GATGGCTGCCGGGCCTCCTGAGAAGCACGACGCAG-TGTGGACTAGAACCCAGAATT
AGTGGCTGCCGGCCCTCCTGAGAAGCAGGA-CAAGGTTTGAACTAGAAGCCAGAATT
GG--------------CCTGAGAAGCCACAAGCAG----------------------
                ****  * **       **
ENSMUSG00000042354
>NM_206826 TCGTCGTG
ATACACAGTTCCCAAAATCCCGGATTCTTTGTGGTGT------TGAGAAGCTGAAATA
----ACAGTTTACAAAAACTC-AACTCGTCGTGACGGTACAAATGAGAAGCTTAAGTA
----ACAGTTTAAAAACACTC-GATTCGTCGTGACGTTACAAATGAGAAGCTTAAGTA
GT--ACAATTT-TAAAACCTC-GATTTTCCGTGCTGCTGCAGAGAAGACACTTCAGTA
    *** **   ***  * *  * *    ***  *         ***  **  * **


Motif 10
ENSMUSG00000041438
>NM_032830 TGACTGAA
GAACAACTCAGAACGTTTT-ACATACAACCGAATACAGATTATTTTTATTTGTAGTGT
AACCAACCTTGTGAGTTTT-ATATATGACTGAATACAAATCATTTTTATTACCAGTGT
GAACAATCTCGGGAGTTTT-ATATATGTCTGAATACAAATCATTTTTATTACCAGTGT
GAACAATTCAGGAAGTTTTCATATACAAATGAACACAGATTATTTTTATTAGCAGTGT
 * ***    *   ***** * ***     *** *** ** *********   *****
ENSMUSG00000041438
>NM_032830 TGACTGAA
TATTTGTAGTGTTATCGTGGTTTTGTAACTGAAGGGGACA----GGTCTATACTTTTG
TATTACCAGTGTCATGGTGGTTGTGTGACTGAAGGGGATGTTT-GACCCA-GTTTTCT
TATTACCAGTGTCATGGTGGTTGTGTGACTGGAGGGGATGTTTGAGCCTA-ATTTTCT
TATTAGCAGTGTTATTGTGGTT-TGTGACTGAAGGGGATG----ATCTTA-AGTTTCA
****   ***** ** ****** *** **** ******           *   ***
ENSMUSG00000038510
>NM_032194 ATTGAAGT
CTAGACGCCCTCTACGAGGCCGTCCCCGGAAGTGGCGTTACGCGCAGCTTCCGGTTCC
ACAGCCG----------GGCTGTCCATTGAAGTGGTATTTCGC---ACTTCCGGCACC
CCCGCCG----------GGCTGTCTAAGGAAGTGGTATTTCGC---ATTTCCGGCACT
-----------------GGCGGCCCCCGGAAGTGGCGTGCCGCGCAAGTTCCGGTCCC
                 *** * *    *******  *  ***     ******  *
ENSMUSG00000054079
>NM_016001 GACTGAAG
CTTAACCAG-----GACATGAATGAGACTGAAGGGTCTAATAAGGGTAGGCAGA-Caa
CTTAACCAGGACACGACATGAATGAGACTGAAGGGTCTAATAAGGGCAGGCAAG-TGA
CTTAACCAGGACATGACATGAATGAGACTGAAGGGTCTAATAAGGGCAGGCAAGTTGA
CTTCACAAG-----GACATGAATGAGACTGAAGGGTCTCATGCGGGCAGGCAAA----
*** ** **     ************************ **  *** *****
```
