# Supplementary material for: Systematic Identification of cis-Regulatory Sequences Active in Mouse and Human Embryonic Stem Cells
Source: PLoS Genet. 2007 Aug 31;3(8):e145. doi: 10.1371/journal.pgen.0030145 (PMC1959362; doi:10.1371/journal.pgen.0030145)

Figure S1. Grskovic et al. 2007

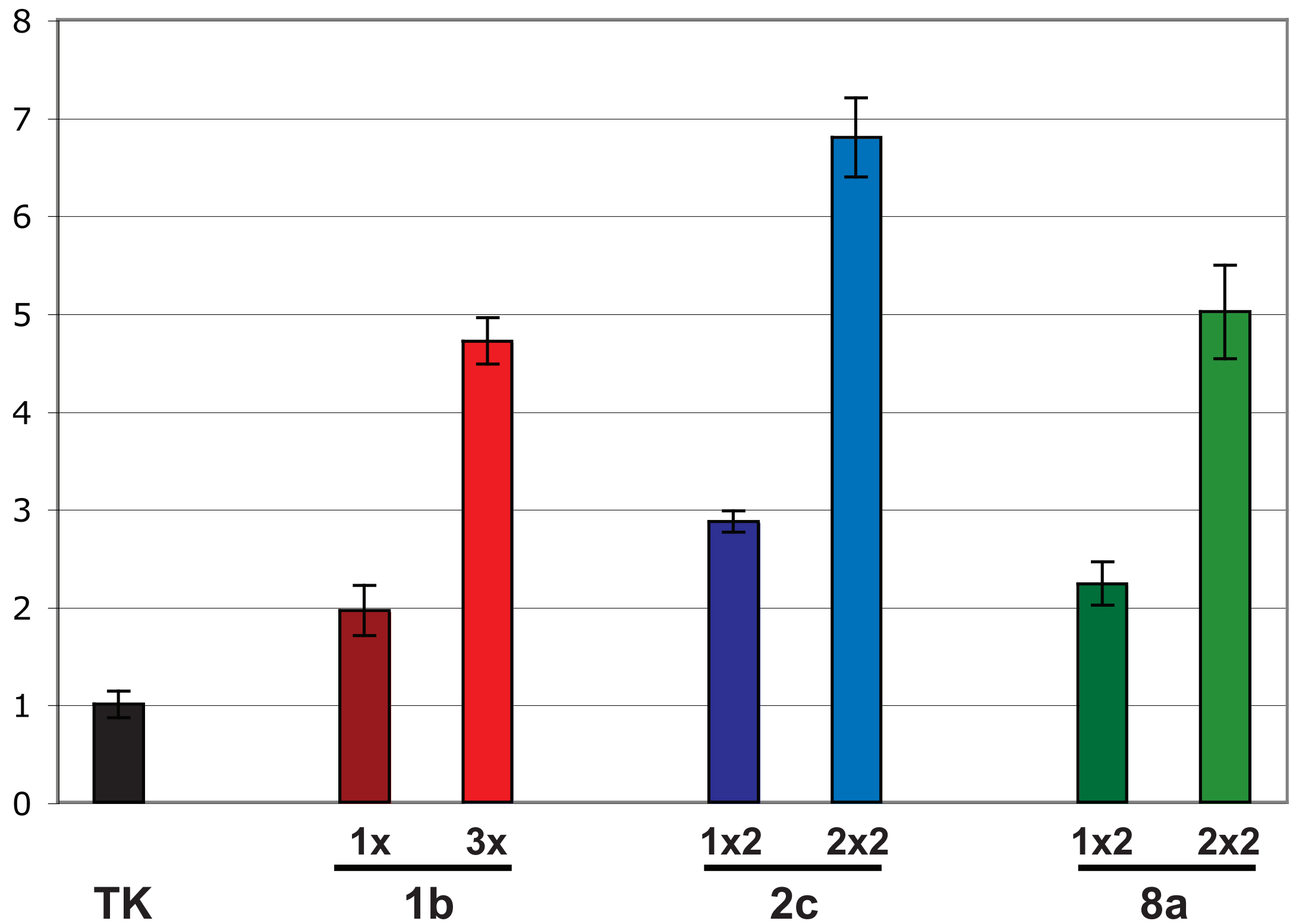

Supplement: Supplementary file 2 [file pgen.0030145.sg001.pdf]
